# Systematic Comparison and Rational Design of Theophylline Riboswitches for Effective Gene Repression

**DOI:** 10.1128/spectrum.02752-22

**Published:** 2023-01-23

**Authors:** Xun Wang, Can Fang, Yifei Wang, Xinyu Shi, Fan Yu, Jin Xiong, Shan-Ho Chou, Jin He

**Affiliations:** a State Key Laboratory of Agricultural Microbiology & Hubei Hongshan Laboratory, College of Life Science and Technology, Huazhong Agricultural University, Wuhan, Hubei, People’s Republic of China; Shenzhen Bay Laboratory

**Keywords:** theophylline riboswitch, biological parts, transcriptional regulation, posttranslational regulation, protein degradation tag

## Abstract

Riboswitches are promising regulatory tools in synthetic biology. To date, 25 theophylline riboswitches have been developed for regulation of gene expression in bacteria. However, no one has systematically evaluated their regulatory effects. To promote efficient selection and application of theophylline riboswitches, we examined 25 theophylline riboswitches in Escherichia coli MG1655 and found that they varied widely in terms of activation/repression ratios and expression levels in the absence of theophylline. Of the 20 riboswitches that activate gene expression, only one exhibited a high activation ratio (63.6-fold) and low expression level without theophylline. Furthermore, none of the five riboswitches that repress gene expression were more than 2.0-fold efficient. To obtain an effective repression system, we rationally designed a novel theophylline riboswitch to control a downstream gene or genes by premature transcription termination. This riboswitch allowed theophylline-dependent downregulation of the TurboRFP reporter in a dose- and time-dependent manner. Its performance profile exceeded those of previously described repressive theophylline riboswitches. We then introduced as the second part a RepA tag (protein degradation tag) coding sequence fused at the 5′-terminal end of the *turborfp* gene, which further reduced protein level, while not reducing the repressive effect of the riboswitch. By combining two tandem theophylline riboswitches with a RepA tag, we constructed a regulatory cassette that represses the expression of the gene(s) of interest at both the transcriptional and posttranslational levels. This regulatory cassette can be used to repress the expression of any gene of interest and represents a crucial step toward harnessing theophylline riboswitches and expanding the synthetic biology toolbox.

**IMPORTANCE** A variety of gene expression regulation tools with significant regulatory effects are essential for the construction of complex gene circuits in synthetic biology. Riboswitches have received wide attention due to their unique biochemical, structural, and genetic properties. Here, we have not only systematically and precisely characterized the regulatory properties of previously developed theophylline riboswitches but also engineered a novel repressive theophylline riboswitch acting at the transcriptional level. By introducing coding sequences of a tandem riboswitch and a RepA protein degradation tag at the 5′ end of the reporter gene, we successfully constructed a simple and effective regulatory cassette for gene regulation. Our work provides useful biological components for the construction of synthetic biology gene circuits.

## INTRODUCTION

Riboswitches are common biological regulatory parts typically located in the 5′ untranslated region (UTR) of mRNAs that alter gene expression in response to small molecule ligands (1, 2). They mainly contain two domains: a ligand-binding domain (aptamer) and an output domain (expression platform) that regulates downstream gene expression (3, 4). Due to their specificity, modular design, simplicity, and ease of implementation, riboswitches provide a promising platform for gene regulation.

To meet the growing demand, more than 60 artificial riboswitches that respond to nonmetabolite ligands, including theophylline, tetracycline, naringenin, caprolactam, and dopamine, have been constructed (5–10). Among them, the theophylline riboswitch is the most studied (11). In 2004, Suess et al. engineered the first theophylline riboswitch by combining the theophylline aptamer with an expression platform, resulting in a functional translational “ON” (TL-ON) riboswitch (5). Shortly thereafter, Desai and Gallivan constructed another TL-ON theophylline riboswitch (12). Later, other researchers developed theophylline riboswitches with different regulatory mechanisms. For example, Ogawa et al. fused the theophylline aptamer to a hammerhead ribozyme to generate a ribozyme-based ON (RZ-ON) riboswitch (13). Fowler et al. obtained the first transcriptional ON (TC-ON) theophylline riboswitch by fluorescence-activated cell sorting (FACS) (14), and Topp and Gallivan further constructed a translational “OFF” (TL-OFF) theophylline riboswitch by inserting a riboswitch coding sequence within the translated region of a gene (15). On top of that, Ceres et al. rationally designed three chimeric riboswitches, each containing the same theophylline aptamer domain but fused to three different expression platforms from *metE*, *yitJ*, and *lysC* riboswitches, to generate three functional transcriptional OFF (TC-OFF) riboswitches (16). Meanwhile, various research teams have extensively screened and optimized the theophylline riboswitch libraries to improve their regulatory efficiencies. To accurately characterize the regulatory efficiencies of riboswitches, the researchers further introduced the concept of the activation/repression ratio, a quantitative relationship between the concentration of a small molecule inducer and the output signal of a biosensor (17). Activation/repression ratios were calculated as the fold change between the maximum and minimum values of the biosensor output signal. For example, Topp et al. screened and constructed six TL-ON theophylline riboswitches termed A to E and E*, capable of inducing gene expression in eight different bacterial species, with activation ranging from 5.0- to 150.0-fold (18). Cui et al. modified the ribosome binding site (RBS) in riboswitch E to generate riboswitch E1, which results in a 6.8-fold activation in Bacillus subtilis (19). Afterwards, Canadas et al. generated four riboswitches based on the sequence of riboswitch E* to provide efficient activation in *Clostridium* (20). Details of the aforementioned riboswitches are shown in Table S1 in the supplemental material.

From the above findings, it can be concluded that theophylline riboswitches have been developed for five distinct regulatory mechanisms, including TL-ON, TC-ON, RZ-ON, TL-OFF, and TC-OFF. Since these theophylline riboswitches originated from different laboratories, their evaluation conditions were somewhat different. Therefore, it is necessary to systematically compare their regulatory efficiencies under the same experimental conditions. To accurately characterize theophylline riboswitches, we focused on two important parameters: the activation/repression ratio and the expression level of the reporter gene in the absence of the inducer theophylline (21). We examined 25 theophylline riboswitches commonly used in bacterial cells, including 17 TL-ON, 3 TC-ON, 1 RZ-ON, 1 TL-OFF, and 3 TC-OFF riboswitches. We investigated these two parameters in Escherichia coli strain MG1655 and found that they differed from those of previous reports. We also compared the data in different E. coli strains and growth media and at different temperatures and found that, with the exception of the TL-ON riboswitches, the other riboswitches were not well regulated. To obtain a more effective regulatory cassette, we employed two strategies: first, we rationally designed and constructed a novel TC-OFF theophylline riboswitch by connecting two riboswitches in tandem to improve their activation/repression ratios; Second, to further lower the protein level, we utilized a protein degradation tag to shorten the half-life of the reporter proteins and thereby achieved lower protein amounts. We also constructed a mathematical model to predict the systemic repression efficiencies at different theophylline concentrations. This work thus provides a new regulatory cassette for bottom-up design of genetic circuits that will facilitate rational engineering of gene expression in synthetic and systems biology.

## RESULTS

### Systematic evaluation of theophylline riboswitches.

**FIG 1:**
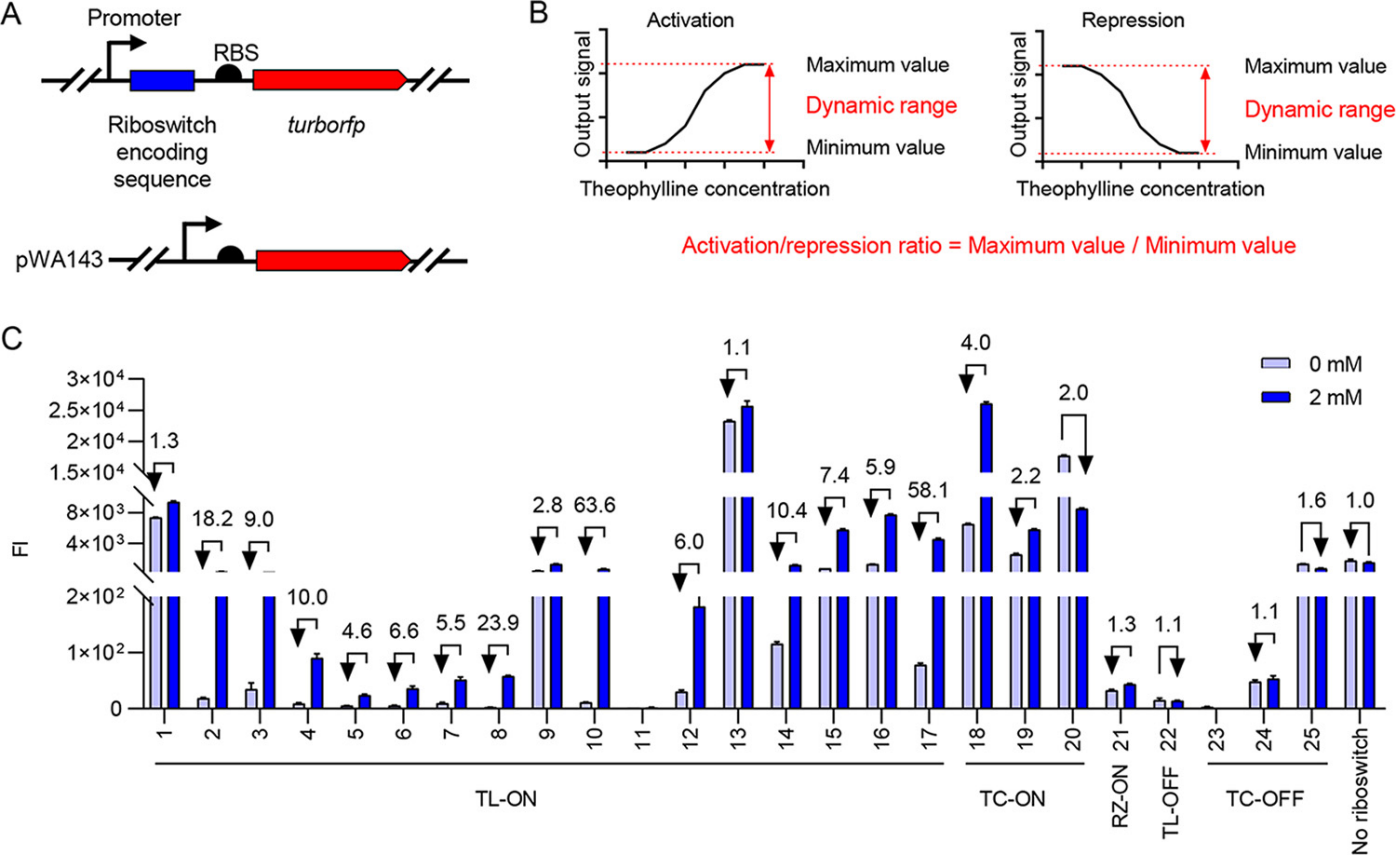
Evaluation of the regulatory efficiencies of various theophylline riboswitches. (A) Design of a plasmid-containing gene circuit to assess the repression efficiencies of theophylline riboswitches. The *turborfp* gene (shown as a red arrow) is controlled by a constitutive promoter (shown as a black arrow). The coding sequences for theophylline riboswitch and RBS are shown as a blue box and black semicircle, respectively. Control plasmid pWA143 contains gene circuit but without the riboswitch coding sequence. (B) Definition of activation/repression ratio and dynamic range. The activation/repression ratio was calculated by dividing the maximum value by the minimum value. In the case of activation, the minimum value is equal to the expression level in the absence of theophylline; in the case of repression, the maximum value is equal to the expression level in the absence of theophylline. “Dynamic range” refers to the maximum and minimum values of the interval. (C) TurboRFP fluorescence intensity (FI) measured in the absence (light blue) and presence (dark blue) of 2 mM theophylline. Theophylline was added at 2 h, and the relative *turborfp* mRNA expression levels or FI was measured at 12 h. Numbers above the column represent activation/repression ratios. Data represent the mean ± standard deviation (SD) from three biological replicates.

To test the performance of each riboswitch, we first compiled the detailed sequences of 25 theophylline riboswitches commonly used in bacteria reported to date by reviewing the literature and classified them into 17 TL-ON (no. 1 to 17), 3 TC-ON (no. 18 to 20), 1 RZ-ON (no. 21), 1 TL-OFF (no. 22), and 3 TC-OFF (no. 23 to 25) riboswitches according to their regulatory mechanisms and effects (see Table S1 in the supplemental material). We then inserted the coding sequences for the different theophylline riboswitches individually upstream of the reporter gene *turborfp* (except TL-OFF riboswitch no. 22, which is in the translated region of *turborfp*), controlled by a strong constitutive promoter, J23100 (Fig. 1A) (22), and constructed 25 different plasmids containing theophylline riboswitch gene circuits (Table S2). We also constructed a pWA143 plasmid containing the reporter gene circuit, but without the riboswitch coding sequence, as a negative control (Fig. 1A). We selected the most representative wild-type E. coli strain, MG1655, as the host strain, and incubated the MG1655-derived strains harboring different plasmids in lysogeny broth (LB) medium at 37°C as the culture condition. We then examined the inhibitory effect of theophylline on the growth of the derived strains and found negligible growth inhibition in LB medium when the theophylline concentration was 2 mM or lower (Fig. S1). Therefore, unless otherwise indicated, all of the following experiments used 2 mM theophylline.

To evaluate these riboswitches, we examined their performance on activation/repression ratios and expression levels in the absence of theophylline. The activation/repression ratio was calculated by dividing the TurboRFP FI at 2 mM theophylline by that at 0 mM theophylline. The expression level in the absence of theophylline refers to TurboRFP FI at 0 mM theophylline. These two parameters together constitute the dynamic range of the riboswitch: that is, the maximum and minimum values that it can regulate (Fig. 1B).

First, we focused on the activation/repression ratios of these riboswitches (Fig. 1C). MG1655-derived strains were grown for 2 h to reach early exponential phase. Theophylline was then added at this time point, and the mRNA expression levels or FI of *turborfp* were measured at 12 h. Most of the 17 TL-ON riboswitches showed 2.2- to 63.6-fold activation, but no. 1 and 13 exhibited less than 2.0-fold activation, and the strain harboring the no. 11 riboswitch exhibited almost no fluorescent signal. Among the 3 TC-ON riboswitches, no. 18 and 19 promoted TurboRFP expression more than 2.0-fold in the presence of theophylline, whereas no. 20 showed the opposite effect under our experimental condition; instead of activating, it repressed gene expression. For the no. 21 riboswitch of RZ-ON, the data showed little effect. It can be seen that among the 21 ON switches described above, including 17 TL-ON and 3 TC-ON switches and 1 RZ-ON switch, no. 10 had the highest activation ratio of 63.6-fold. We also analyzed 4 OFF switches, of which the TL-OFF no. 22 riboswitch was only 1.1-fold effective. Three TC-OFF riboswitches (no. 23, 24, and 25) were also tested. Only no. 25 showed a 1.6-fold difference in gene repression, while the other two showed little change in TurboRFP FI under theophylline induction.

Next, we compared expression levels in the absence of theophylline and found that they were quite different (Fig. 1C). Among the ON riboswitches, the expression levels of TurboRFP ranged from as low as 2.5 arbitrary units (AU) (TL-ON no. 8) to as high as 23,000 AU (TL-ON no. 13) without theophylline addition, meaning that they were nearly 10,000 times different. In the OFF riboswitches, there was also a large difference in the expression levels of TurboRFP in the absence of the inducer theophylline. For example, the highest (TC-OFF no. 25) and lowest (TL-OFF no. 22) levels were 1,300 and 15 AU, respectively. Given the limited activation/repression ratios of most riboswitches, these differences cannot be ignored. For example, if both riboswitches activated gene expression up to 10.0-fold and if the expression level of one is 100 AU and that of the other is 1,000 AU without the inducer theophylline, they were expressed at dynamic ranges of 100 to 1,000 AU and 1,000 to 10,000 AU, respectively, which could lead to a large difference in the biological output. That said, when we describe the activation/repression ratios of riboswitches, we should also pay attention to their expression levels in the absence of theophylline.

Finally, we tested the performance of the aforementioned 25 riboswitches under different conditions, including different E. coli host strains, growth media, and temperatures. Although the degrees of activation/repression were different, it is worth noting that for most riboswitches, the dynamic range didn’t change much (Fig. S2). For example, for the no. 3 TL-ON riboswitch, the activation ratio of strain JM101 in LB medium at 37°C was only 1.1, while that of strain DH5α reached 9.6 in LB medium at 25°C. Although far different, the TurboRFP FI was still within the dynamic range of 10 to 300 AU. The same was true for other riboswitches, such as the no. 13 TL-ON riboswitch, whose dynamic range was consistently within 20,000 to 40,000 AU, regardless of the conditions. Therefore, testing riboswitches under various conditions allowed us to more accurately assess their regulatory efficiencies. Notably, the no. 10 TL-ON riboswitch showed excellent results, with over 12.0-fold activation under all testing conditions. Its expression levels fluctuated in the range of 3 to 76 AU in the absence of theophylline (Fig. S2). Therefore, this riboswitch was the best player among all ON riboswitches. On the contrary, all OFF riboswitches performed poorly, with repression ratios of less than 2.0-fold. For example, the no. 25 TC-OFF riboswitch was ineffective in the catabolite repression medium used (SOC medium [see Materials and Methods]) and at 25°C.

### Rational design of TC-OFF theophylline riboswitch.

**FIG 2:**
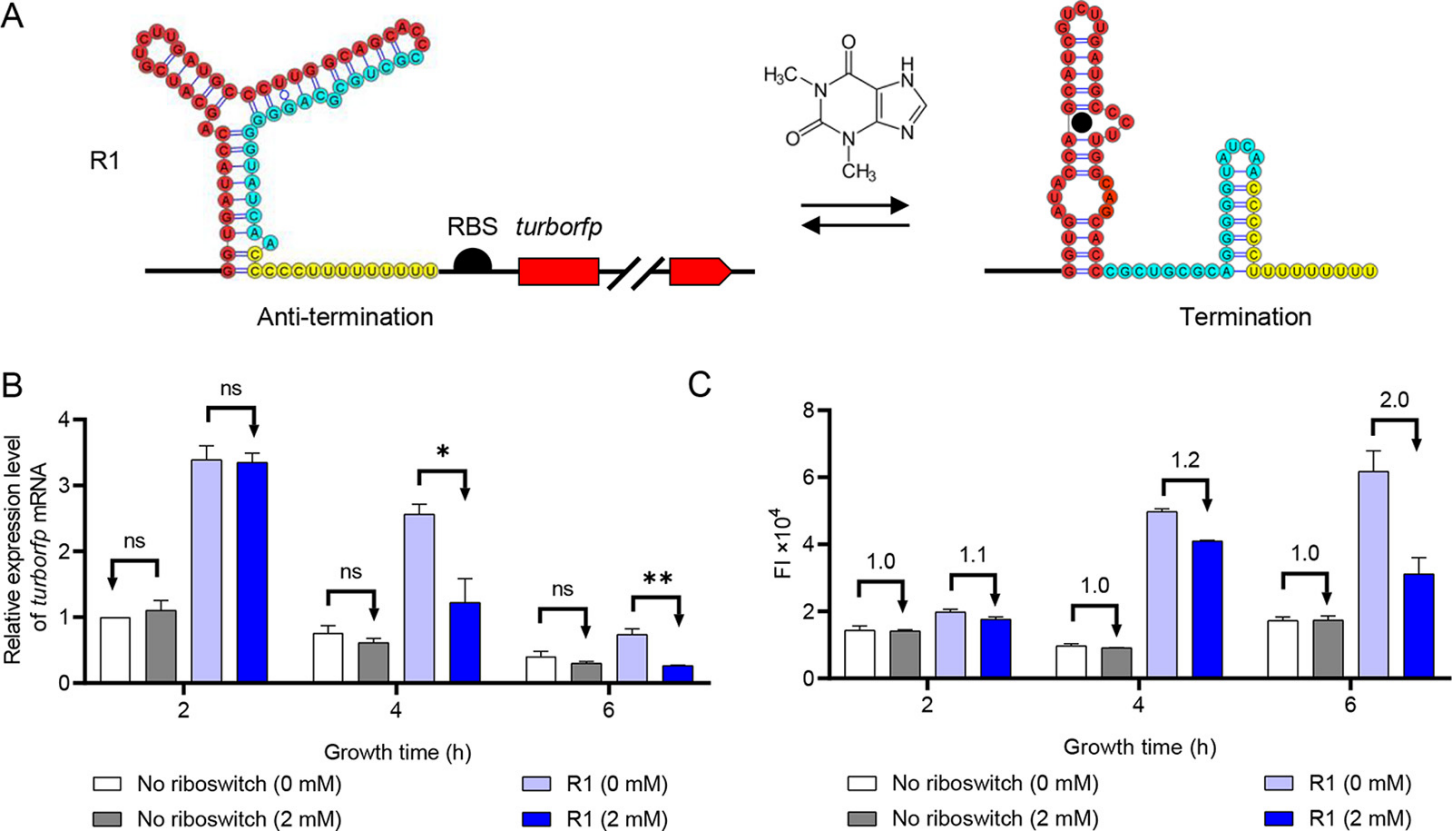
Evaluation of the regulatory efficiencies of the rationally designed TC-OFF theophylline riboswitches. (A) Design strategy for theophylline-dependent riboswitch R1 to control transcription. The theophylline aptamer (red) was fused to an intrinsic transcription terminator (cyan and yellow). Sequences modified from the previous riboswitch B are marked in yellow. The RBS sequence (black semicircle) and the open reading frame of the reporter gene *turborfp* are located downstream of this construct. In the absence of theophylline, intrinsic terminator formation is inhibited, resulting in transcription readthrough and *turborfp* expression. Upon binding of theophylline (black solid circle), an intrinsic terminator is formed and transcription is prematurely stopped, resulting in repression of *turborfp* expression. (B) Relative expression levels of *turboRFP* mRNA measured in the absence (white and light blue) and presence (gray and dark blue) of 2 mM theophylline. 16S rRNA was used as an internal control. Data were subjected to one-way analysis of variance (ANOVA) using the Bonferroni test. ns, not significant (*P* > 0.05). Asterisks indicate significant differences: *, 0.01 < *P* < 0.05; **, 0.001 < *P* < 0.01. (C) TurboRFP FI measured in the absence (white and light blue) and presence (gray and dark blue) of 2 mM theophylline. Numbers above the column represent activation/repression ratios. Data represent the mean ± SD from three biological replicates.

Since none of the OFF riboswitches showed more than a 2.0-fold repression ratio, we decided to redesign and reconstruct a repressive riboswitch. Among the riboswitches for transcriptional regulation, there are two types of regulation based on (i) intrinsic terminators and (ii) Rho-dependent terminators (23, 24). Intrinsic terminators are sequences in the nontemplate DNA strand that, when transcribed into RNA, form a GC-rich hairpin structure followed by a U-rich tract in the RNA-DNA hybrid (25). It causes dissociation of the elongation complex without the assistance of auxiliary transcription regulators. Transcriptional control through intrinsic termination is a more conserved, relatively simple, and efficient regulatory mechanism compared to translation- and ribozyme-based regulation (26). Considering the advantages of an intrinsic terminator-based transcriptional control, we sought to develop an intrinsic terminator-based TC-OFF theophylline riboswitch. Riboswitch B (the no. 6 TL-ON switch in this work) was previously reported to activate protein translation (18). In the absence of theophylline, riboswitch B folds into an OFF state with the RBS sequestered in the secondary structure. When theophylline binds to an aptamer, the RBS gains access to the 16S rRNA of the ribosome to initiate translation (18). Interestingly, we found that the RBS sequence AGGGGGU is G-rich, which represents exactly half of the intrinsic transcription terminator hairpin sequence. We speculated that a complete intrinsic transcription terminator could be constructed if the sequence downstream of RBS was replaced by the other half of the terminator. Therefore, we changed the sequence CAAGAUG to CCCCCUU and added another 7 U residues (UUUUUUU) downstream of it. The terminator is thus composed of a 7-bp hairpin stem, a 4-bp loop, and a stretch of 8 U residues (Fig. 2A). We named this new riboswitch R1 and expected it to transcriptionally repress expression. Since the translation initiation codon AUG in riboswitch B was deleted in the new construct, we added an RBS and an AUG codon downstream of the riboswitch to initiate TurboRFP translation. The corresponding plasmid was named pWA131 (Fig. S3 and Table S2).

To test whether R1 is functional *in vivo*, MG1655/pWA131 (test strain) or MG1655/pWA143 (control strain) was used and the relative *turborfp* mRNA expression levels or FI were measured at 2, 4, and 6 h. The results showed that the expression of *turborfp* mRNA in the test strain decreased by 2.1- and 2.8-fold at 4 and 6 h after theophylline addition, respectively, compared to the case without theophylline addition. Meanwhile, the relative *turborfp* mRNA expression levels of the control strains were not significantly changed regardless of the presence or absence of theophylline (Fig. 2B). Trends in TurboRFP FI for testing or control strains were consistent with changes in relative *turborfp* mRNA expression levels. After theophylline addition, the TurboRFP FI decreased by 1.2- and 2.0-fold at 4 and 6 h in the test train, respectively, compared to the strains without theophylline addition (Fig. 2C). Likewise, there was no significant change in the TurboRFP FI of the control strain at 0 or 2 mM theophylline (Fig. 2C). Therefore, the addition of theophylline resulted in decreased mRNA and protein levels, demonstrating that the TC-OFF theophylline riboswitch we constructed was indeed effective.

### Improving riboswitch performance.

**FIG 3:**
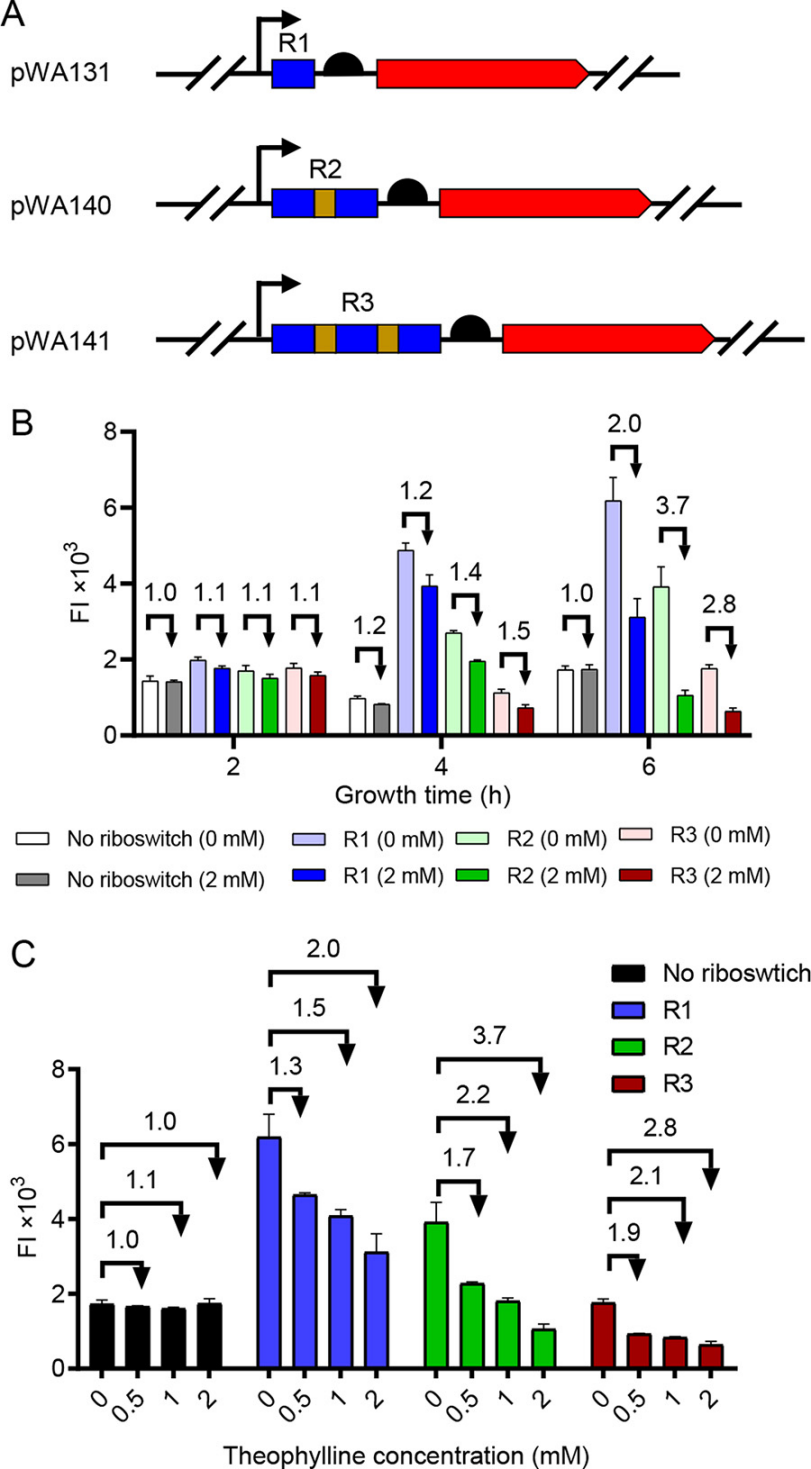
Evaluation of the regulatory efficiencies of tandem TC-OFF theophylline riboswitches at different theophylline concentrations. (A) Schematic of plasmids containing the engineered gene circuit controlled by a tandem theophylline riboswitch. Coding sequences of the promoter (black arrow), riboswitches (blue box), linker (brown box), RBS (black semicircle), and *turboRFP* (red arrow) are shown. R1 represents one riboswitch, R2 represents two riboswitches in tandem, and R3 represents three riboswitches in tandem. (B) TurboRFP FI measured in each strain harboring a plasmid with different tandem riboswitches (R1, R2, and R3) in the absence and presence of 2 mM theophylline; (C) TurboRFP FI of each strain grown in LB medium supplemented with 0, 0.5, 1.0, and 2 mM theophylline. FI was measured 6 h after theophylline addition. Numbers above the columns represent activation/repression ratios. Data represent the mean ± SD from three biological replicates.

However, we noticed that the repression ratio of the riboswitch was not large enough. To address this issue, we found previous literature showing that riboswitches in tandem can achieve a greater repression ratio (27, 28). Therefore, we linked two or three theophylline riboswitch coding sequences via a 13-bp linker (28) sequence upstream of *turborfp*, to generate the pWA140 (R2) and pWA141 (R3) plasmids, respectively (Fig. 3A). Detailed sequences and derivative strains are listed in Fig. S3. At 4 h, strains harboring R2 and R3 repressed TurboRFP expression by 1.4- and 1.5-fold in the presence of theophylline; at 6 h, these values increased to 3.7- and 2.8-fold, respectively (Fig. 3B). From the data presented above, it could be seen that R2 gave the highest repression ratio at 6 h. We also found that increasing the number of tandem riboswitches in the test strain reduced the FI of TurborRFP in the absence of theophylline from about 61,800 AU (R1), to 39,100 AU (R2), to 17,600 AU (R3) at 6 h. After theophylline addition, the corresponding TurboRFP FI also decreased from 31,200 AU (R1), to 10,600 AU (R2) to 6,400 AU (R3) (Fig. 3B).

The response of tandem riboswitches to different concentrations of theophylline was also tested. We measured the FI 6 h after the addition of 0, 0.5, 1.0, and 2 mM theophylline. We found 1.3- 1.7-, and 1.9-fold repression ratios of R1, R2, and R3 at the theophylline concentration of 0.5 mM, respectively (Fig. 3C), and no repression was observed in the control strain MG1655/pWA143 (“No riboswitch” in Fig. 3C). With the increase of theophylline concentration, the repression ratios of R1, R2, and R3 also increased accordingly. At concentrations of 1.0 and 2.0 mM theophylline, R2 exhibited 2.2- and 3.7-fold higher repression efficiencies than R1 and R3, respectively (Fig. 3C). Considering the repression ratio and expression level in the absence of theophylline, we concluded that R2 performed the best among the three.

### Mathematical model of R2-mediated quantifiable repression.

**FIG 4:**
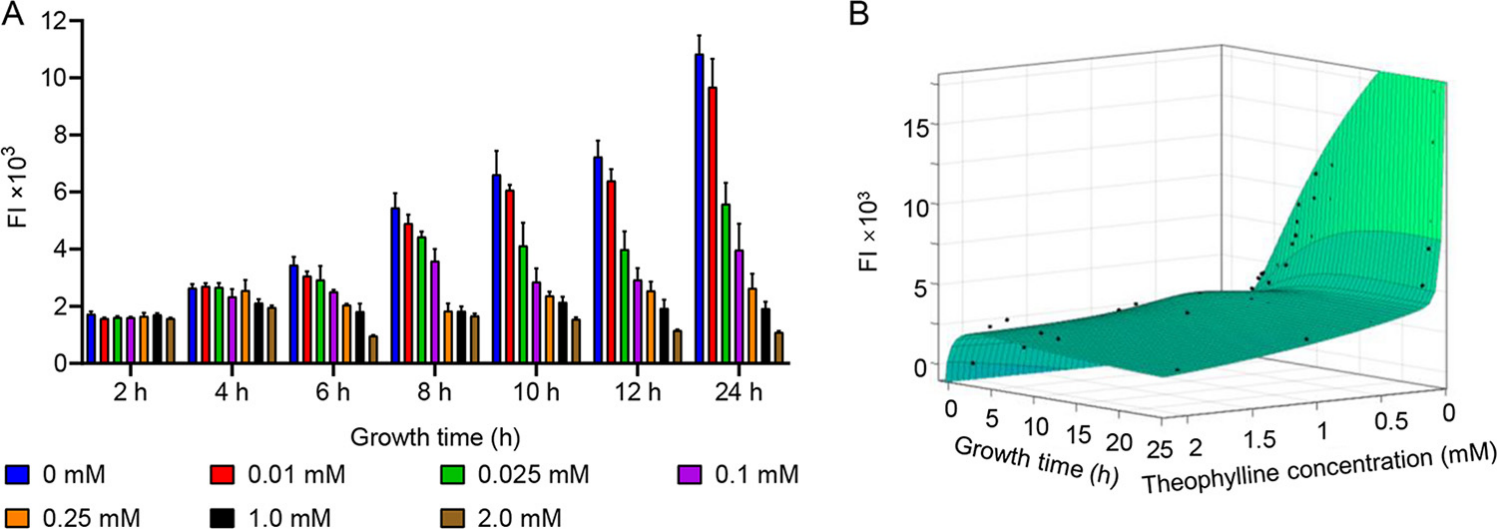
Comparison of experimental measurements with model predictions. (A) TurboRFP FI measured from 2 to 24 h in the pWA140 (R2)-containing strain at various theophylline concentrations from 0 to 2.0 mM. Data represent mean ± SD of three biological replicates. (B) Mathematic model of R2 based on all data measured at different theophylline concentrations and growth times. The *x* axis represents growth time, the *y* axis represents the theophylline concentration, and the *z* axis indicates the TurboRFP FI. Data represent the mean ± SD from three biological replicates.

To validate our observations and provide predictability, we set out to construct a mathematic model of the regulatory efficiency of R2 at different concentrations of theophylline and different times. For this, the parameters of the model become an important component. These parameters must be derived from experimental data and represent biological properties. Thus, MG1655/pWA140 was grown in LB medium supplemented with theophylline at final concentrations of 0, 0.01, 0.025, 0.1, 0.25, 1.0, and 2.0 mM. Samples were taken every 2 h from 2 to 12 h, and the TurboRFP FI was measured (Fig. 4A). When the growth medium was supplemented with 2 mM theophylline, the highest repression level was 10.0-fold at 24 h (Fig. 4A).

To generate the model, we applied several assumptions and simplifications. (A detailed derivation and description of the model are provided in the supplemental Materials and Methods.) Finally, a simplified equation considering *turboRFP* dynamic equilibrium was proposed (equation 9 in the supplemental Materials and Methods). As the simulations showed, our model agreed well with the experiment results (*R*^2^ = 0.9755) (Fig. 4B). If a user wants to know the TurboRFP FI at a specific time point and theophylline concentration, the mathematical model can be used for calculation. Conversely, a user can infer the appropriate concentration of theophylline in the medium from the TurboRFP FI at a specific time according to the model.

In the results presented above, we examined the regulatory efficiency of the theophylline riboswitches on TurboRFP expression based on the FI. To verify whether the FI is a reliable parameter for evaluating the expression level of TurboRFP, we compared the relationship between the TurboRFP amount and its FI. We chose MG1655/pBRPcon (pBRPcon is a pBRPlac-derived plasmid [without *turborfp*] in which the *lac* promoter was replaced by the promoter J23100 [Table S2]) as the control strain and MG1655/pWA140 as the test strain. We incubated these two strains at various theophylline concentrations for 12 h and separated total proteins by sodium dodecyl sulfate-polyacrylamide gel electrophoresis (SDS-PAGE) (Fig. S4A). The results showed that the amount of TurboRFP followed the trend of FI (Fig. S4B). The results presented above indicated that it is reasonable to use FI as a measure for the amount of expressed protein.

### Robustness of R2 under various conditions.

**FIG 5:**
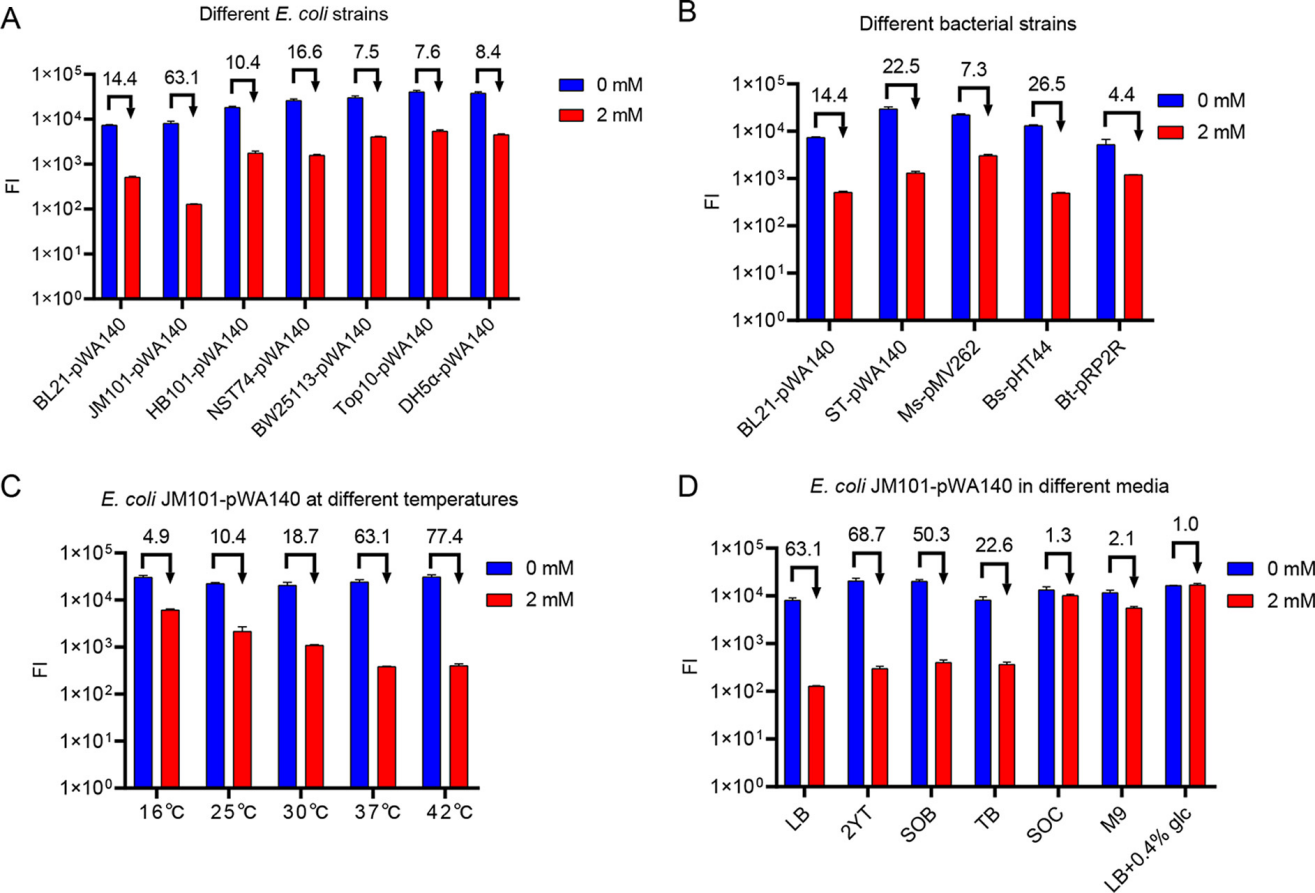
Evaluation of the regulatory efficiencies of R2 in different strains and culture conditions. (A) Regulation of TurboRFP expression by R2 in different E. coli strains; (B) regulation of TurboRFP expression by R2 in different bacterial species; (C) regulation of TurboRFP expression by R2 in the E. coli JM101/pWA140 strain grown at different temperatures; (D) regulation of TurboRFP expression by R2 in the E. coli JM101/pWA140 strain grown in different media. Theophylline (2 mM) was added at 2 h, and FIs were measured at 24 h. Numbers above the columns represent activation/repression ratios. Data represent the mean ± SD from three biological replicates.

We then tested the robustness of R2 in different E. coli stains harboring pWA140, including BL21/pWA140, JM101/pWA140, HB101/pWA140, NST74/pWA140, BW25113/pWA140, Top10/pWA140, and DH5α/pWA140. In all strains, the presence of R2 did show a significant repression effect on TurboRFP expression. Among them, R2 in JM101/pWA140 provided the highest repression ratio (Fig. 5A). The robustness of R2 was also tested in five different bacterial species widely used in genetic engineering, including phylum *Proteobacteria* E. coli BL21/pWA140, Salmonella enterica serovar Typhimurium SL1344/pWA140, phylum *Actinobacteria* Mycobacterium smegmatis MC^2^155/pMV262, phylum *Firmicutes* B. subtilis 168/pHT44, and Bacillus thuringiensis BMB171/pRP2R (Tables S2 and S3). The results showed that R2 provided more than 7.0-fold repression of TurboRFP expression in most strains (Fig. 5B).

Given that temperature is also an important factor affecting riboswitch performance (29), we selected the JM101/pWA140 strain, which had the best regulatory efficiency, to test the riboswitch performance at five different temperatures ranging from 16°C to 42°C. Indeed, R2 exhibited significant repression at all tested temperatures and was more efficient at 42°C than at 16°C, with a repression ratio of 77.4-fold versus 4.9-fold (Fig. 5C). We attributed these results to the higher mobility of the RNA structure at elevated temperatures (30).

R2 function was also measured in different growth media and found to exhibit good regulatory efficiencies in seven tested media except SOC (Fig. 5D). By examining the composition of the media, we found that the presence of glucose in SOC might prevent the repressive function of R2. To test this speculation, we added 0.4% glucose to LB medium and subsequently measured TurboRFP FI in the JM101/pWA140 strain. As expected, R2 was inactive in this medium. These data suggested a certain correlation between glucose concentration and theophylline transport. In conclusion, our rationally designed TC-OFF theophylline riboswitch could control gene expression in different bacterial species and growth media and at different temperatures, making it a useful tool for repressing gene expression.

### Dual transcriptional and posttranslational control R2-RepA tag system further reduces gene expression.

**FIG 6:**
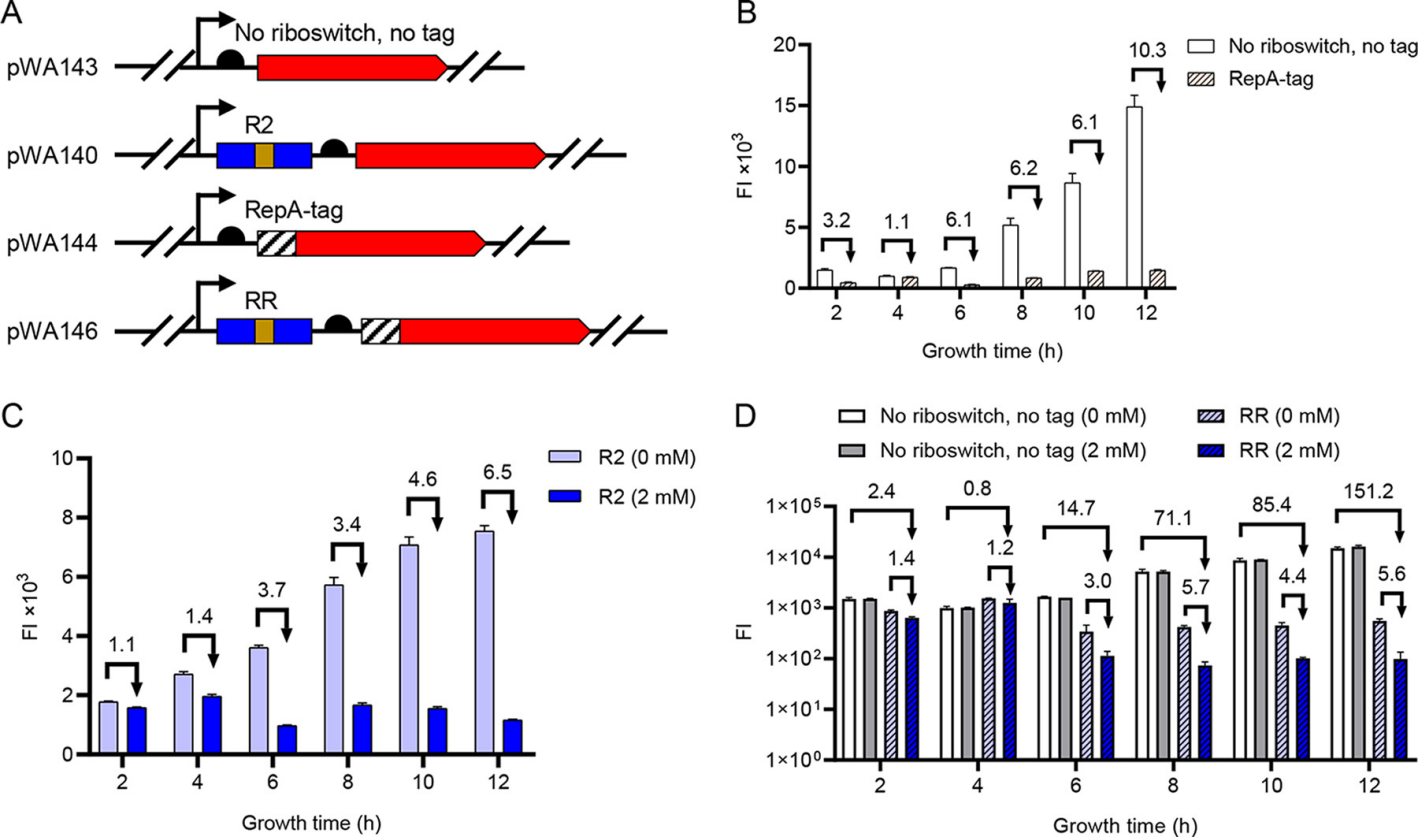
Regulation of TurboRFP expression by the RR system. (A) Schematic of plasmids containing the coding sequences of R2, RBS, or the RepA tag upstream of *turborfp*. pWA143 was used as a control plasmid without regulatory sequences upstream of *turborfp.* pWA140, pWA144, and pWA146 represent the plasmids containing the tandem riboswitch coding sequence, the RepA tag coding sequences, and both of these parts, respectively. (B) Regulation of TurboRFP expression by the protein degradation RepA tag at different growth phases. The numbers above the columns represent the TurboRFP FI of the “No riboswitch, no tag” treatment divided by that of the “RepA-tag” treatment. (C) Regulation of TurboRFP expression by R2 at 0 or 2.0 mM theophylline from 2 to 12 h. The numbers above the columns represent the TurboRFP FI at 0 mM theophylline divided by that at 2 mM theophylline. (D) Regulation of TurboRFP expression by the RR system at 0 and 2.0 mM theophylline from 2 to 12 h. Numbers above the column represent repression ratios. All data above represent the mean ± SD from three biological replicates.

Through the experiments described above, we found that the regulatory efficiency of R2 in E. coli JM101/pWA140 was as high as 77.4-fold, while the repression efficiency in the widely used wild-type MG1655/pWA140 strain was only 10.0-fold at the maximum repression level at 24 h (Fig. 4A). To further reduce TurboRFP expression in MG1655/pWA140, we introduced a second gene repression part into the system: a protein degradation tag (RepA tag), which consists of 15 amino acids (NQSFISDILYADIES) and directs the target protein to the housekeeping ClpAP protease (31, 32). This part can be used to shorten the half-life of proteins, thereby reducing protein levels. We considered it a promising regulatory tool because it is located at the N terminus of the protein, so the RepA tag coding sequence could be easily integrated with the riboswitch coding sequence as a single regulatory cassette. We anticipated that the RepA tag-red fluorescent protein (RFP) fusion protein could serve as a substrate for ClpAP degradation (Fig. 6A); the detailed sequence is listed in Fig. S3. The repression ratio of the RepA tag was examined from 2 to 12 h. As shown in Fig. 6B, the FI of RepA tag-RFP was indeed reduced in MG1655/pWA144 (“RepA-tag” in Fig. 6B) compared to untagged TurboRFP in MG1655-pWA143 (“no riboswitch, no tag” in Fig. 6B), but RepA tag-RFP remained prominently expressed throughout the growth period. The expression level of RepA tag-RFP was the lowest at 6 h, and the FI gradually accumulated from 8 h to 12 h. One possible reason is that in the stationary phase, the amounts of proteins targeted for degradation by proteases increased, and they competed for a limited number of proteases, resulting in a prolonged half-life of RepA tag-RFP (33). We also tested the expression of TurboRFP in the MG1655/pWA140 strain from 2 to 12 h. In the absence of theophylline, TurboRFP FI increased over time, whereas with 2 mM theophylline, FI decreased. The repression ratio increased from 1.4-fold at 4 h to 6.5-fold at 12 h (Fig. 6C). It is worth mentioning that the MG1655/pWA140 strain still exhibited obvious TurboRFP expression after 6 to 12 h.

Next, the R2-RepA tag (RR) system was inserted upstream of *turborfp* (pWA146), in which two theophylline riboswitch coding sequences in tandem were fused to the RepA tag coding sequence (Fig. 6A and Fig. S3). We then measured the regulatory efficiencies of the RR system in medium with 0 or 2 mM theophylline. We observed continuous expression of TurboRFP from 2 to 12 h in the MG1655/pWA143 strain (“no riboswitch, no tag, 0 mM” in Fig. 6D). Addition of theophylline did not affect the expression of TurboRFP (“no riboswitch, no tag, 2 mM” in Fig. 6D). Introduction of the RR system reduced the expression of TurboRFP in MG1655/pWA146 (“RR, 0 mM” in Fig. 6D). Addition of 2 mM theophylline further reduced TurboRFP expression in MG1655/pWA146 (“RR, 2 mM” in Fig. 6D). At 12 h of incubation, the repression efficiencies of RepA tag and R2 were 26.9- and 5.6-fold, respectively, which together constituted up to 151.2-fold repression (“RR, 2 mM” versus “no riboswitch, no tag, 0 mM” in Fig. 6D). From the above results, it can be seen that combining transcriptional regulation and posttranslational regulation can effectively repress gene expression.

### RR system located in chromosome repressed *lacZ* expression.

**FIG 7:**
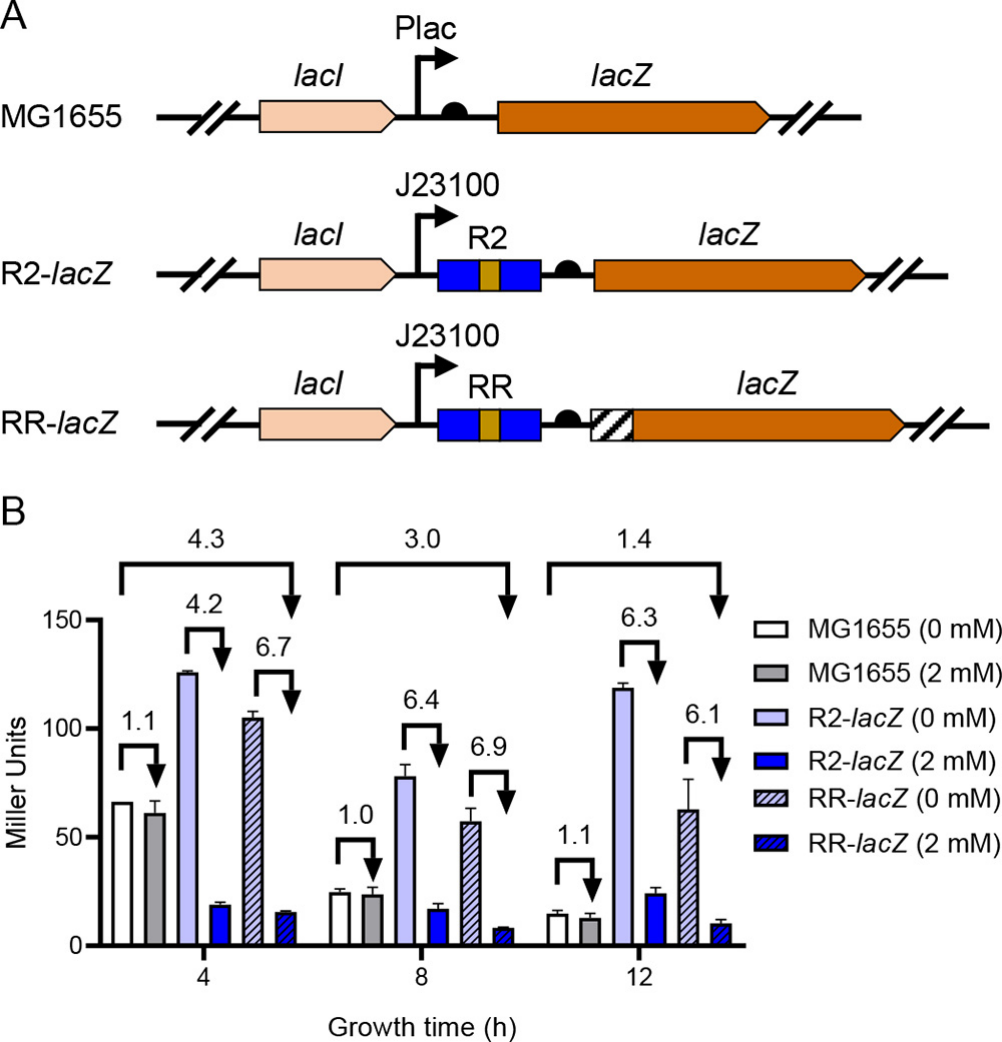
Regulation of LacZ expression by R2 or the RR system located in the chromosome. (A) Schematic diagram of strains containing the coding sequences of R2 (R2-*lacZ*) or RR system upstream of *lacZ* (RR*-lacZ*); (B) regulation of LacZ expression by R2 or the RR system at 0 or 2.0 mM theophylline at 4, 8, and 12 h. All data above represent the mean ± SD from three biological replicates.

For the application of the RR system, we examined the regulation of this cassette by inserting it upstream of *lacZ* on the chromosome. We constructed an RR-*lacZ* strain, in which the 123-bp intergenic region between *lacI* and *lacZ* was replaced by the RR system (Fig. 7A). Also, we constructed the R2-*lacZ* strain, which is a derivative of the RR-*lacZ* strain without the RepA tag coding sequence (Fig. 7A).

In strain MG1655, we detected a gradual decrease in β-galactosidase activity from 66 Miller units to 14 Miller units after 4 to 12 h of incubation. Addition of theophylline had no effect on β-galactosidase activity (“MG1655, 0 mM” versus “2 mM” in Fig. 7B). Introduction of R2 or the RR system (R2-*lacZ* and RR-*lacZ* strains) increased the overall expression of LacZ compared to MG1655. In the R2-*lacZ* strain, β-galactosidase activity was reduced about 4.0- to 6.0-fold when grown under 2 mM theophylline compared to without theophylline addition (Fig. 7B). Similar results were obtained in the RR-*lacZ* strain. This indicated that the theophylline riboswitch can still exert repression effects on chromosome. However, the RR-*lacZ* strain (under 2 mM theophylline) showed only 4.3-, 3.0-, and 1.4-fold repression at 4, 8, and 12 h compared to MG1655 without theophylline addition (Fig. 7B). We shall discuss the specific reasons for this in the Discussion session. In conclusion, the RR system located on the chromosome can still repress the expression of functional genes.

## DISCUSSION

### A highly efficient dual gene expression regulatory system.

In this study, we systematically evaluated 25 theophylline riboswitches used in bacteria. We determined their activation/repression ratios and expression levels in various strains and growth media and at various temperatures in the absence of theophylline. Among the five regulatory mechanisms, the TL-ON riboswitches were optimized best, especially riboswitch no. 10, as it showed the highest activation ratio and the lowest expression level in the absence of theophylline. However, none of the OFF riboswitches showed more than a 2.0-fold repression ratio, so we redesigned and reconstructed a novel repressive theophylline riboswitch by combining transcriptional and posttranslational regulation. We did successfully construct a dual gene expression regulatory RR system based on tandem riboswitches and the RepA protein degradation tag. The base length of the RR system is only 218 bp and did not require expression of exogenous proteins. The protein level could be repressed by simply inserting this new regulatory cassette upstream of the protein-coding sequence. Therefore, these works provide the basis for a more rational selection of theophylline riboswitches.

### Possible reasons for the poor performances of the previously described theophylline riboswitches.

In this section, we tried to find the reason for the poor performance of the previously described theophylline riboswitches. The activation/repression ratio was less than 2.0-fold for riboswitches TL-ON no. 1 and 13, TC-ON no. 20, RZ-ON no. 21, TL-OFF no. 22, and TC-OFF no. 23, 24, and 25. We first evaluated their full-length riboswitch structures via the RNAfold website (34) and found that, among these riboswitches, two TL-ON riboswitches, no. 1 and 13, had the most unstable secondary structures, with minimum free energies (MFEs) of only −0.13 and −0.11 kcal/mol/bp, respectively. This means that, in the absence of theophylline, the RBS and start codon were often accessible for translation initiation, resulting in significant leaky expression. We also compared the structural differences between full-length riboswitches and riboswitches with only the aptamer domain, which were constrained to form efficient ligand-binding folds. RZ-ON riboswitch no. 21, TL-OFF riboswitch no. 22, and TC-OFF no. 23, 24, and 25 riboswitches showed no secondary structure changes in either state. Therefore, we speculated that theophylline has little effect on maintaining the theophylline-bound secondary structure of these RNAs, resulting in no regulatory effect. We also calculated the free energy differences between theophylline-bound and -unbound states of these riboswitches. The free energies of the TC-ON no. 20 riboswitch in the theophylline-bound and -unbound states were −15.9 and −38.5 kcal/mol, respectively. Thus, the free energy difference for no. 20 was −22.6 kcal/mol, which deviated significantly from the binding energy of the aptamer/theophylline complex (−8.86 kcal/mol) (35). Therefore, this RNA molecule did not appear to be folding into the conformation claimed by the authors.

We also noticed that the switching efficiency of specific riboswitches varied with host strain, growth medium, growth phase, and temperature. The discrepancy between our experimental conditions and those reported in the literature may also be one of the reasons for the suboptimal results reported for the theophylline riboswitches.

### Applications of the RR system for dual transcriptional and posttranslational control.

Compared to the numerous strategies for achieving high gene expression levels in E. coli, relatively few repression systems were available (36, 37). To date, three systems are commonly used in bacterial cells; the tetracycline repression system (Tet-off system), clustered regularly interspaced short palindromic repeats interference (CRISPRi), and small RNA (sRNA)-meditated gene repression. While useful in a large number of applications, these systems have limitations. The Tet-off and CRISPRi systems require additional expression of exogenous proteins TetR and Cas, which increases system complexity and imposes a metabolic burden on bacteria (38, 39). Compared to these three repression systems, the current RR system had several advantages: (i) no exogenous protein expression is required, (ii) the RR system is very short, only 218 bp in length, and (iii) theophylline was found to be a nonmetabolic inducer and can be considered functionally orthogonal to E. coli metabolism (40). For these reasons, our system can be used as a general approach for repressing gene expression.

However, the RR system also had certain limitations. First, the degradation of RepA tag-labeled protein is relatively weak in exponential phase cells due to the insufficient amount of degradation complexes, so it is difficult to achieve efficient repression in exponential-phase bacteria (41). Second, like the N-terminal signal peptide or N-terminal His tag, there are cases that may impair the function of the target protein (42), the RepA tag may only be applied to some proteins, and this needs to be further verified by more experiments. Third, the repression efficiency of the RepA tag is related to the half-life of the target protein. The half-life of proteins in E. coli varies from minutes to a few days (43), and the RepA tag may be more suitable for controlling bacterial proteins with long half-lives: for example, it can shorten the half-life of green fluorescent protein (GFP) from more than 1 day to 15 min (44, 45). We believe that the RepA tag produces different repression ratios for different proteins, and it exhibits less pronounced repression on proteins with short half-lives. This may also be the reason why the repressive efficiency of the RepA tag upstream LacZ is only 2.0-fold. Fourth, the repressive efficiency of RR system depends on promoter activity. We observed an overall higher expression of LacZ in the RR-*lacZ* strain than in MG1655. This is due to the higher activity of the J23100 promoter than the original *lac* promoter. Therefore, in practical applications, the activity of the promoter also needs to be considered to achieve the best regulatory effect. In addition, it has been reported that plasmid copy number affects the regulation of riboswitches (46), and our study is currently limited to the medium-copy-number plasmid pBR322. For high- or low-copy plasmids, further investigation is required.

## MATERIALS AND METHODS

### Plasmid construction.

In E. coli and *S.* Typhimurium, reporter plasmids were constructed using pBR322 as the parent plasmid (47, 48). In M. smegmatis, B. thuringiensis, and B. subtilis, plasmids pMV261 (49, 50), pRP0122 (28), and pHT43 (51) were used, respectively. Except for pHT43, the transcription of *turborfp* along with its 5′-UTR regulatory element was carried out under a strong constitutive promoter, J23100. In pHT43, gene expression was controlled by an isopropyl-β-d-thiogalactopyranoside (IPTG)-inducible Pgrac promoter. The primer pairs (see Table S4 in the supplemental material) used for plasmid construction were synthesized by Tianyi Huiyuan (Wuhan, Hubei, China). All plasmids generated in this study (Table S2) were assembled using the Hieff Clone Plus multi one-step cloning kit (Yeasen, Shanghai, China), and confirmed via sequencing (Quintarabio, Wuhan, Hubei, China). The bacterial cells were transformed with the constructed plasmids via a calcium chloride (CaCl_2_) method to produce corresponding derivative strains (Table S3).

### Bacteria and culture conditions.

E. coli DH5α was used for all cloning experiments. If not indicated otherwise, E. coli MG1655 cells were transformed with the resulting plasmids for fluorescence measurement. E. coli R2-*lacZ* and RR-*lacZ* strains were used for β-galactosidase assay. The construction of the R2*-lacZ* and RR*-lacZ* strains was performed essentially as reported previously (52). E. coli NST74 (53), BL21, HB101, JM101, BW25113, and Top10, *S.* Typhimurium SL1344 (54), *M. smegmatis* MC^2^155 (55), B. thuringiensis BMB171 (24, 56), and B. subtilis 168 were employed as hosts for the tests of the riboswitch regulatory efficiencies (Table S3). The derived strains of E. coli, *S.* Typhimurium, and B. thuringiensis were grown in LB medium (10 g/L tryptone, 5 g/L yeast extract, 10 g/L NaCl) and M. smegmatis in 7H9 medium (4.9 g/L 7H9 broth, 0.2% glycerol, 0.05% Tween 20). For B. subtilis-derived strains, 2× yeast extract-tryptone (2×YT) medium (16 g/L tryptone, 10 g/L yeast extract, 5 g/L NaCl) was used for cultivation. Super optimal broth with catabolite repression (SOC) medium is composed of 2% tryptone, 0.5% yeast extract, 10 mM NaCl, 2.5 mM KCl, 10 mM MgCl_2_, and 20 mM glucose. To measure the regulatory efficiency of R2, JM101/pWA140 was grown in super optimal broth (SOB) medium (20 g/L tryptone, 5 g/L yeast extract, 0.5 g/L NaCl, 0.186 g/L KCl, 0.95 g/L MgCl_2_, with the pH of the medium adjusted to 7.0 with 5 N NaOH), terrific broth (TB) medium (20 g/L tryptone, 24 g/L yeast extract, 4 mL/L glycerol, 2.2 g/L KH_2_PO_4_, 9.4 g/L K_2_HPO_4_), and M9 medium (12.8 g/L Na_2_HPO_4_·7H_2_O, 3 g/L KH_2_PO_4_, 0.5 g/L NaCl, 1.0 g/L NH_4_Cl, 2 mM MgSO_4_, 0.05 mM CaCl_2_, 0.4% glucose). When necessary, ampicillin, kanamycin, or spectinomycin was added to the culture at the final concentrations of 100, 50, or 100 μg/mL, respectively. If not otherwise indicated, strains were grown in 250-mL shake flasks (50 mL medium per flask) on a rotary shaker (200 rpm) at 37°C (Ruihua, Wuhan, Hubei, China).

For fluorescence measurement, individual colonies were picked and grown overnight in 5 mL LB medium with 100 μg/mL ampicillin. This culture was diluted 100.0-fold to inoculate 50 mL of fresh medium and allowed to grow to early exponential phase (2 h after inoculation, an optical density at 600 nm [OD_600_] of ~0.5), at which point theophylline was added to the medium at the concentrations indicated. Cells were allowed to grow for different times to measure their FI as indicated in the figure legend.

For protein quantitation and SDS-PAGE analysis of proteins, test strain MG1655/pWA140 was grown at 37°C in an orbital shaker at 200 rpm in LB medium supplemented with 0, 0.025, or 0.25 mM theophylline and 100 μg/mL ampicillin. As a control, MG1655/pBRPcon was grown in LB medium supplemented with 100 μg/mL ampicillin.

For the β-galactosidase assay, the MG1655 and RR-*lacZ* strains were grown at 37°C in an orbital shaker at 200 rpm in LB medium with or without 2 mM theophylline supplementation. Samples were taken at 4, 8, and 12 h for the determination of β-galactosidase activities.

### RNA extraction, cDNA synthesis, and RT-qPCR.

For reverse transcription-quantitative PCR (RT-qPCR) experiments, 2-mL samples from E. coli strains were collected. Total RNA was extracted as previously described (57). First-strand cDNA was synthesized using the PrimeScript RT reagent kit with gDNA Eraser (TaKaRa Biomedical Technology, Beijing, China) according to the manufacturer’s instructions. Specific primer pairs (Table S4) for amplification of *turborfp* and the reference gene 16S rRNA were designed using the online tool provided by GenScript (https://www.genscript.com/tools/real-time-pcr-tagman-primer-design-tool) (Table S4). During design, primer pairs were balanced for melting temperatures and cross-dimers were avoided where possible. The amplification efficiency of each primer set was tested. For the test, cDNA was diluted by 1.0-, 10.0-, 100.0-, 1,000.0-, 10,000.0-, and 100,000.0-fold, and the diluted cDNA was used as a template for the subsequent RT-qPCR. RT-qPCR assays were performed using Hieff UNICON Power qPCR SYBR green master mix (Yeasen, Shanghai, China). Each PCR (20 μL) contained 10 μL of 2× SYBR mix, 0.4 μL PCR forward primer (10 μM), 0.4 μL PCR reverse primer (10 μM), 1 μL template cDNA, and 8.2 μL RNase-free water. The reactions were performed in Bio-Rad CFX96 real-time PCR machine (Bio-Rad Laboratories, TX, USA). The following cycling protocol was used: an initial denaturing step at 95°C for 30 s, followed by an amplification cycle of 95°C for 10 s, followed by 60°C for 30 s, with the cycle number set to 40, and the amplification completed with a product dissociation cycle. Amplification curves were obtained, and the slope was calculated by the lm formula (58) in R version 4.2.2. The efficiency of each primer pair was calculated from the slope using the following formula (59):

$$\text{efficiency}\;\left(\%\right)=(10^{\frac{-1}{\text{slope}}}-1)\times 100$$

The amplification efficiencies of *turborfp* and 16S rRNA were 96.4% and 97.0%, respectively.

For relative quantification of *turborfp* mRNA, RT-qPCRs were performed according to the procedure described above. Amplification curves and melting curves were illustrated by CFX Maestro software 2.3 (Bio-Rad Laboratories, TX, USA). The threshold cycle (*C_T_*) determination mode was set to a single threshold. For data analysis, technical and biological triplet data were obtained. The comparative 2^−ΔΔ^*^CT^* method was used to calculate the relative fold changes of *turborfp* mRNA in the samples (60), and the expression was normalized with the internal control 16S rRNA. The expression of each sample is presented as mean ± SD. The ΔΔ*C_T_* values were subjected to one-way analysis of variance (ANOVA) using the Bonferroni test. GraphPad Prism 9.0 was used for one-way ANOVA and graph plotting.

### Fluorescence measurement.

The OD_600_ and FI of each sample were measured using Infinite M200 Pro multimode microplate reader (Tecan Group, Ltd., Männedorf, Switzerland). One milliliter of each sample was taken, of which 500 μL of the culture was diluted 2 times or more with LB medium until the OD_600_ was less than 0.4 and used to measure the OD_600_, and another 500 μL of the culture was used to measure its FI in a 96-well microplate (Sangon Biotech, Shanghai, China). Fluorescence measurements were carried out at an excitation wavelength of 553 nm, and the emission fluorescence was taken at 593 nm. Background fluorescence was measured by titrating appropriate concentrations of theophylline into cells carrying the parental plasmid pBRPcon and calculating the OD_600_ normalized fluorescence. The value of background fluorescence is ~1,450 AU per OD unit. It includes the fluorescence of bacterial autofluorescence and the fluorescence of LB medium; the fluorescence value of LB medium is ~1,300 AU. After background elimination, the FIs of the riboswitch-containing plasmids were then normalized to the respective cell growth (OD_600_). All experimental results were obtained with three biological replicates.

### Protein quantitation and SDS-PAGE analysis of proteins.

Three milliliters of cells was harvested at 12 h by centrifugation at 16,000 × *g* for 1 min. For protein quantitation, the collected cells were resuspended in 1 mL 1× phosphate buffer saline (pH 7.4) with 500 mM NaCl. The cells were lysed using an ultrasonic homogenizer (Scientz, Ningbo, Zhejiang, China). Sonication was performed on ice for 3 × 1 min at 50% amplitude, pulsing at 3 s on/1 s off. The total amount of protein was quantified by a bicinchoninic acid (BCA) protein quantification kit (Yeasen, Shanghai, China). The protein assay working reagent of BCA was prepared by mixing 50 volume of reagent A (sodium carbonate, sodium bicarbonate, bicinchoninic acid and sodium tartrate in 0.1 M sodium hydroxide) with 1 volume of reagent B (4% CuSO4). Two hundred microliters of BCA working solution was pipetted onto the wells of a 96-well plate, and 25 μL of samples was added, giving a BCA working solution/sample ratio of 8:1. The plates were quickly placed in the reader, shaken for 30 s with a smooth motion before reading, and were gently mixed for 30 s at 37°C using Infinite M200 Pro multimode microplate reader (Tecan Group, Ltd., Männedorf, Switzerland). The light transmission at the 562-nm wavelength was monitored. The subsequent data were exported to GraphPad Prism, and the averages of the replicates were plotted versus concentrations. SDS-PAGE was performed with 5% (wt/vol) stacking gels and 12% (wt/vol) separating gels, and proteins were visualized by Coomassie blue R-250 staining.

### β-Galactosidase assay.

The β-galactosidase specific activities were determined and converted to Miller units as previously described (61). Specifically, 1 mL of each cell culture was used to measure the OD_600_, and then 500 μL of bacterial cells was centrifuged (16,000 × *g* for 1 min) and resuspended in 1 mL of Z buffer (60 mM Na_2_HPO_4_, 40 mM NaH_2_PO_4_, 10 mM KCl, 1 mM MgSO_4_, 50 M β-mercaptoethanol [pH 7.0]). One hundred microliters of chloroform and 100 μL 0.1% SDS were added to each sample and vigorously mixed for 10 s. Samples were incubated at 28°C for 5 min, and 0.2 mL of 4 mg/mL *o*-nitrophenyl-β-d-galactopyranoside (ONPG) in 0.1 M phosphate buffer (60 mM Na_2_HPO_4_, 40 mM NaH_2_PO_4_ [pH 7.0]) was added to each sample to start the reaction. When samples changed color to yellow, the reactions were stopped by the addition of 0.5 mL of 1 M Na_2_CO_3_ and time elapsed since the addition of ONPG was recorded. Samples were centrifuged at 16,000 × *g*, and the *A*_420_ values of the supernatants were measured. β-Galactosidase activity was calculated according to the formula:

$$\text{Miller units}=1,000\times \frac{A_{420}-1.75\times A_{525}}{A_{600}\times (T-T_{0})\times V}$$

where *T* is the time of the reaction in minutes and *V* is the volume of the culture used in milliliters. The β-galactosidase specific activity was normalized by the total protein content. The values shown represent the average from three independent experiments.

### Mathematical model.

The mathematical model was generated in MATLAB R2019b. The equations used were based on the law of mass action that describes the biomolecular interactions. A detailed derivation and description of the model is provided in the Results section and in the supplemental material (62, 63).
