# Supplementary material for: Systematic Comparison and Rational Design of Theophylline Riboswitches for Effective Gene Repression
Source: Microbiol Spectr. 2023 Jan 23;11(1):e02752-22. doi: 10.1128/spectrum.02752-22 (PMC9927458; doi:10.1128/spectrum.02752-22)
Supplement: Supplemental file 1 — Supplemental material. Download spectrum.02752-22-s0001.pdf, PDF file, 1.0 MB [file spectrum.02752-22-s0001.pdf]

Supplementary files *for*

Systematic comparison and rational design of theophylline riboswitches for effective gene repression

Xun Wang*[†], Can Fang[†], Yifei Wang, Xinyu Shi, Fan Yu, Jin Xiong, Shan-Ho Chou & Jin He*

State Key Laboratory of Agricultural Microbiology & Hubei Hongshan Laboratory, College of Life Science and Technology, Huazhong Agricultural University, Wuhan, Hubei 430070, P. R. China

Contents

1. Supplementary Materials and Methods 2
2. Supplementary Figures S1-S4
Supplementary Figure S1
Growth of *Escherichia coli* MG1655.......... 6
Supplementary Figure S2
Measurement of the regulatory effect of various theophylline riboswitches 7
Supplementary Figure S3
DNA Sequences upstream of *turboRFP* 14
Supplementary Figure S4
The relationship between TurboRFP amount and its FI.......... 15
3. Supplementary Figures S1-S4
Supplementary Table S1
A complete list of theophylline riboswitches published to date. 16
Supplementary Table S2
Plasmids used in this study. 25
Supplementary Table S3
Strains used in this study. 27
Supplementary Table S4
Primers used in this study. 30
4. Supplementary References 37

Supplementary Materials and Methods

Mathematical model

To generate this model, we applied the following assumptions and simplifications:

1) **The cellular environment is homogenous.** Our goal is to obtain a simplified model that is more suitable for computational analysis, but avoid oversimplification that would result in a lack of biological relevance. Although the physiological states of cells at different growth stages are different, to simplify the model and avoid introducing too many parameters, we assumed that within the scope of the model discussion, the various biological components in each cell are the same. The physiological state and gene expression of a single cell are the same in the same culture system, and the components between mother cells and daughter cells are the same. The learning parameters remain basically stable. During data preprocessing, TurboRFP fluorescence intensity (FI) referred to the FI of individual cell.
2) **Theophylline enters the cell by passive diffusion.** Previous studies of theophylline uptake in *Escherichia coli* showed that the intracellular concentration is only 7 nM when the external concentration is 10 μM (1). This suggests that theophylline does not have an active intracellular transport system. We assume that theophylline enters the cell by passive diffusion. Using *Te* to denote the extracellular theophylline concentration and *Ti* to denote the intracellular theophylline concentration, then: $T_e \xrightarrow{D \cdot V_c} T_i$ and $T_i \xrightarrow{D} T_e$. The diffusion coefficient $D = \frac{SP_n}{V_{Cell}}$ min^{-1}, which is jointly determined by the cell surface area $S = 4\pi r^2$, the cell membrane permeability $P_n = 3 \cdot 10^{-3} \mu m$ ·min^{-1} and the *E. coli* volume $V_{cell} = 1.1 \cdot 10^{-9} \mu L/cell$. $V_c = \frac{V_{Cell}}{V_{ext}}$ is the ratio of the intra- and extracellular volumes ratio. Taken together, the relationship regarding the change of theophylline concentration inside and outside the cell can be expressed as (Equation 1 and 2):

$$\frac{dT_e}{dt} = D \cdot T_i - D \cdot V_c \cdot T_e \quad ①$$

$$\frac{dT_i}{dt} = D \cdot V_c \cdot T_e - D \cdot T_i \quad ②$$

3) **The transcription of *turborfp* is tightly controlled by theophylline riboswitch.** The expression of *turborfp* was regulated by changing the concentrations of theophylline through the theophylline riboswitch. Promoter activity was not affected by theophylline. The changes of *turborfp* mRNA degradation rate due to different 5'-UTR sequences were not considered in this model.
4) **There are two types of transcripts containing theophylline riboswitch: full length transcripts and pre-terminated transcripts. The two transcripts have different rates of synthesis and degradation.** The full-length transcript is 869 bp in length, containing *turborfp* mRNA with two theophylline riboswitches in tandem. The pre-terminated transcript is 177 bp in length and contains two theophylline riboswitches in tandem. It would take different times to synthesize them due to their different length. Since translation significantly affects mRNA degradation, we believed that degradation rates also differed between full-length transcripts and pre-terminated transcripts. Pre-terminated transcripts containing two theophylline riboswitches in tandem were divided into two types, one that binds one molecule of theophylline and the other that binds two molecules of theophylline. Their synthesis and degradation rates are the same.
5) **Not all RNAs with theophylline riboswitch sequences could bind theophylline, and not all theophylline riboswitches that bind theophylline could trigger transcriptional termination**. The described phenomenon could lead to different kinds of theophylline riboswitches containing RNA. To clearly

distinguish and quantitatively describe these RNAs, we introduced the partition coefficient δ. In the formula, it was subdivided into δ1 to δ5, which respectively refer to different partition coefficients. See the table below for details.

6) **Different concentrations of theophylline (0-2 mM) had little effect on intracellular metabolism and cell viability.** *E. coli* has been reported to be unable to degrade theophylline (2). Meanwhile, the added concentration range of theophylline did not affect the growth of *E. coli*. We assumed that theophylline (0-2 mM) would have minimal effects on intracellular metabolism and cell viability.

The interaction between mRNA and intracellular theophylline can produce the following types of mRNA: (i) full-length mRNA. It is divided into two types: The first one is not bound by theophylline, is a major contributor to intracellular TurboRFP; the second one is bound by theophylline, and is a minor contributor to intracellular TurboRFP, an occupant of theophylline. (ii) truncated mRNA: transcription termination event generates truncated mRNA. Such mRNAs can be divided into mRNAs that bind to one copy of theophylline leading to transcription termination and mRNAs that bind to two copies of theophylline leading to transcription termination. Those mRNAs have different degradation rates. The following biophysical model can be constructed (Equation 3):

$$DNA \xrightarrow{S} mRNA \begin{cases} +T_i \xrightarrow{K_1} mRNA\_T_i \begin{cases} \cdot\, \delta_1 + T_i \xrightarrow{K_2} mRNA\_T_i\_T_i \begin{cases} \cdot\, \delta_4 \xrightarrow{dm_2+\mu} \emptyset \\ \cdot\, \delta_5 \xrightarrow{dm_1+\mu} \emptyset \end{cases} \\ +T_i \xleftarrow{K_{-2}} mRNA\_T_i\_T_i \\ \cdot\, \delta_2 \xrightarrow{dm_2+\mu} \emptyset \\ \cdot\, \delta_3 \xrightarrow{dm_1+\mu} \emptyset \end{cases} \\ +T_i \xleftarrow{K_{-1}} mRNA\_T_i \\ \xrightarrow{dm_1+\mu} \emptyset \end{cases} \quad ③$$

Based on the above model, the correlation functions for the dynamic changes of each type of mRNA within a single cell of *E. coli* can be obtained as follows (Equation 4-7):

$$\frac{dmRNA}{dt} = S \cdot DNA - (dm_1 + \mu) \cdot mRNA - mRNA \cdot T_i \cdot K_1 + mRNA\_T_i \cdot K_{-1} \quad ④$$

$$\frac{dmRNA\_T_i}{dt} = mRNA \cdot T_i \cdot K_1 - mRNA\_T_i \cdot K_{-1} - \delta_1 \cdot mRNA\_T_i \cdot T_i \cdot K_2 + mRNA\_T_i\_T_i \cdot K_{-2} - \delta_2 \cdot (dm_2 + \mu) \cdot mRNA\_T_i - \delta_3 \cdot (dm_1 + \mu) \cdot mRNA\_T_i \quad ⑤$$

$$\frac{dmRNA\_T_i\_T_i}{dt} = \delta_1 \cdot mR\,NA_{T_i} \cdot T_i \cdot K_2 - mRNA_{T_{i_{T_i}}} \cdot K_{-2} - \delta_4 \cdot (dm_2 + \mu)\, mRNA\_T_i\_T_i - \delta_5 \cdot (dm_1 + \mu) \cdot mRNA\_T_i\_T_i \quad ⑥$$

$$\frac{dn}{dt} = \mu \cdot n \cdot \left(1 - \frac{n}{Kmax}\right) \quad ⑦$$

For the function characterizing the change of each substance by the given parameters the figure can be plotted as shown in the figure below (Hypothetical models of intracellular mRNA and protein levels). Because the overall mRNA level is also correlated with the number of bacteria, we also need to take into account the bacterial cell number, using the classical logistic growth curve of *E. coli* as a model parameter. The main purpose of the production of these figures is not to precisely determine the content of each substance in individual cells of

E. coli, but to make a basic simulation of the trend of these substances, which can be used to further express the relationship between the three substances: theophylline, TurboRFP and time. The simulation results are in excellent agreement with the expected results, indicating that the model is reasonable.

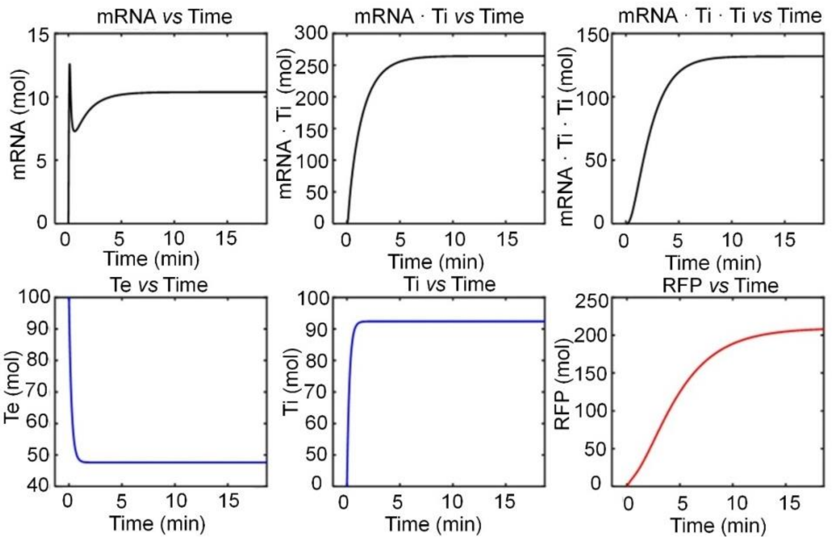

Hypothetical models of intracellular mRNA and protein levels

The intracellular $mRNA_{total} = mRNA + mRNA\_T_i + mRNA\_T_i\_T_i$, only $mRNA$, $\delta_2 \cdot mRNA\_T_i$ 、 $\delta_5 \cdot mRNA\_T_i$ are involved in the synthesis of TurboRFP. So, formulating the rate of protein synthesis as p and the rate of degradation as dp, the correlation function for the dynamic change of TurboRFP in a single cell of *E. coli* can be obtained as follows (Equation 8):

$$\frac{dRFP}{dt} = p \cdot mRNA + p \cdot \delta_3 \cdot mRNA\_T_i + p \cdot \delta_5 \cdot mRNA\_T_i\_T_i - dp \cdot RFP \quad ⑧$$

Based on the above differential equations, a simplified equation considering TurboRFP dynamic equilibrium was proposed (Equation 9):

$$DNA \xrightarrow{K_R} mRNA \left\{ \begin{array}{c} +T_i \xrightarrow{K_1} mRNA\_T_i \left\{ \begin{array}{c} \cdot\, \delta_1 + T_i \xrightarrow{K_2} mRNA\_T_i\_T_i \left\{ \begin{array}{l} \cdot\, \delta_4 \xrightarrow{dm_2+\mu} \emptyset \\ \cdot\, \delta_5 \xrightarrow{dm_1+\mu} \emptyset \end{array} \right. \\ +T_i \xleftarrow{K_{-2}} mRNA\_T_i\ \_T_i \\ \cdot\, \delta_2 \xrightarrow{dm_2+\mu} \emptyset \\ \cdot\, \delta_3 \xrightarrow{dm_1+\mu} \emptyset \end{array} \right. \\ +T_i \xleftarrow{K_{-1}} mRNA\_T_i \\ \xrightarrow{dm_1+\mu} \emptyset \end{array} \right\} \xrightarrow{p} RFP \xrightarrow{dp} \emptyset \quad ⑨$$

The above process can be described as: TurboRFP coding sequence (DNA) is transcribed to *turborfp* mRNA (mRNA). mRNA is bound to 1 molecule of theophylline ($mRNA\_T_i$), or degraded/diluted; $mRNA\_T_i$ can be further bound by 1 molecule of theophylline ($mRNA\_T_i\_T_i$), or degraded/diluted; $mRNA\_T_i\_T_i$ ends up as full-length transcript (dm_1) or truncated transcript (dm_2) form. The full-length transcript (dm_1) is translated into turboRFP and eventually degraded/diluted.

E .coli cells were grown in LB medium supplemented with theophylline to final concentrations of 0, 0.001, 0.01, 0.025, 0.05, 0.1, 0.25, 0.5, 1 and 2 mM. Samples were taken every two hours from 2 to 12 h. The

TurboRFP FI was measured as described in Materials and Methods. The single cell FI was calculated as follows:

$$Single\ cell\ FI_0 = \frac{TurboRFP\ FI}{OD_{600} \cdot 0.8 \times 10^9}$$

The parameters were solved by replacing t, T_i, and TurboRFP with actual experimental data using the "curve fitting" tool in MATLAB 2019b and plotted in Figure 4B. The X-axis stands for growth time, Y-axis stands for theophylline concentration, Z1-axis stands for TurboRFP FI. The correlation coefficient is 0.9755, which shows a good correlation.

**Parameters**	**Description**	**Unit**
C_n	Plasmid copy number	molecules
D	Diffusion coefficient	min^{-1}
dm_1	mRNA degradation rate	min^{-1}
dm_2	mRNA_Ti and mRNA_Ti_Ti degradation rate	min^{-1}
dp	TurboRFP degradation rate	min^{-1}
K_1	The binding constant of theophylline and mRNA	adimensional
K_{-1}	The dissociation constant of theophylline and mRNA	adimensional
K_2	The binding constant of theophylline and mRNA_Ti	adimensional
K_{-2}	The dissociation constant of theophylline and mRNA_Ti	adimensional
$Kmax$	Maximum growth capacity	cells
K_R	mRNA transcription rate	min^{-1}
$mRNA\_T_i$	mRNA which binds a copy of theophylline	adimensional
$mRNA\_T_i\_T_i$	mRNA which binds two copies of theophylline	adimensional
p	mRNA translation rate	min^{-1}
S	mRNA synthesis constant	min
T_e	Extracellular theophylline	molecules
T_i	Intracellular theophylline	molecules
V_c	Cell volume ($1.1x10^{-9}$ µL)/External volume ($1x10^{-3}$ µL)	adimensional
μ	Dilution rate	min^{-1}
δ	The partition coefficient	adimensional
∅	Degradation products	adimensional

Supplementary Figure S1

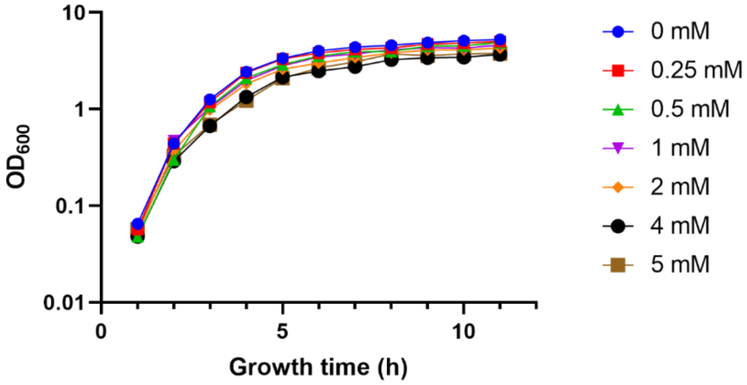

Supplementary Figure S1. Growth of *E. coli* MG1655.

Growth of *E. coli* MG1655 after adding different concentrations of theophylline in LB medium.

Supplementary Figure S2

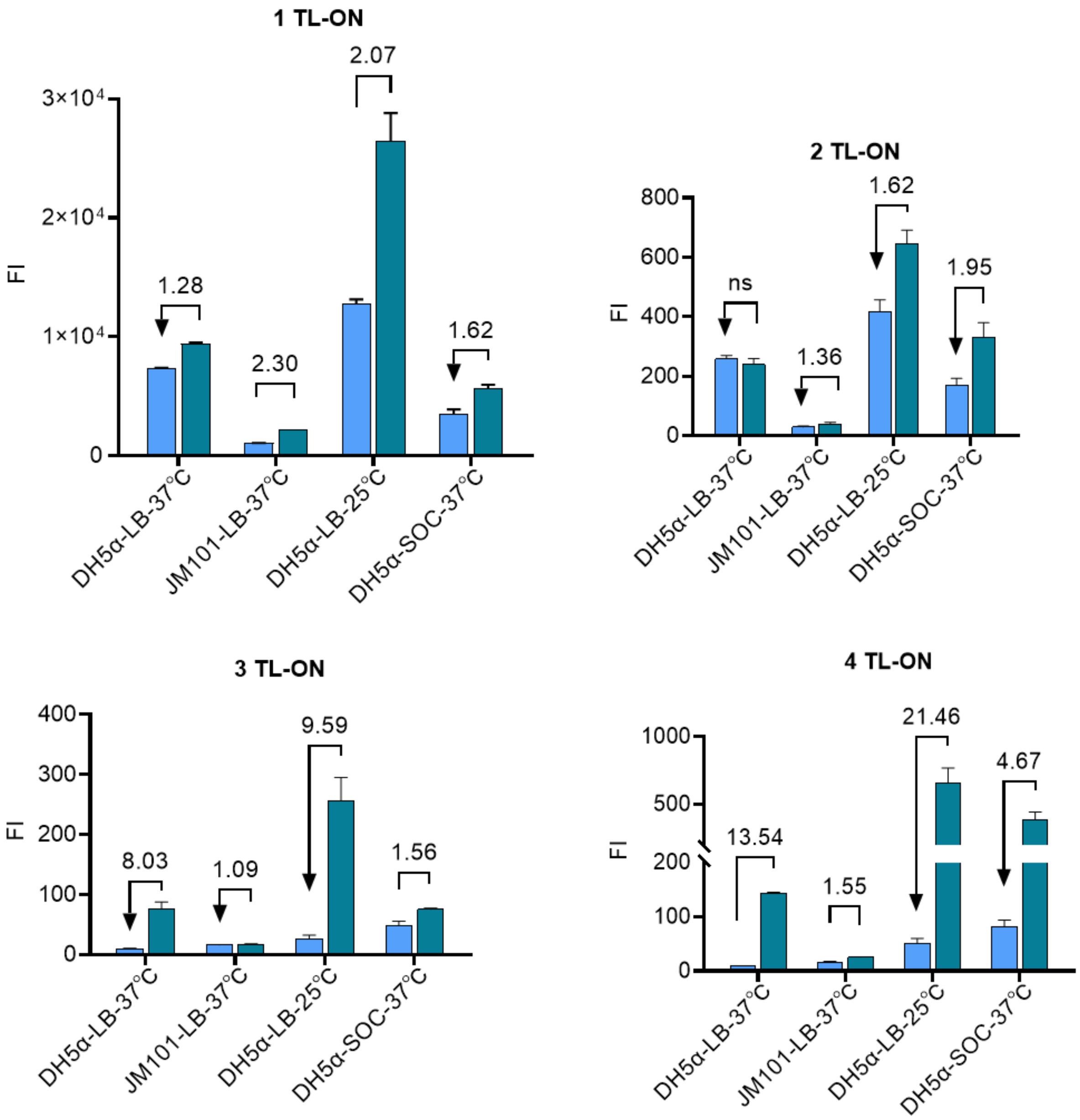

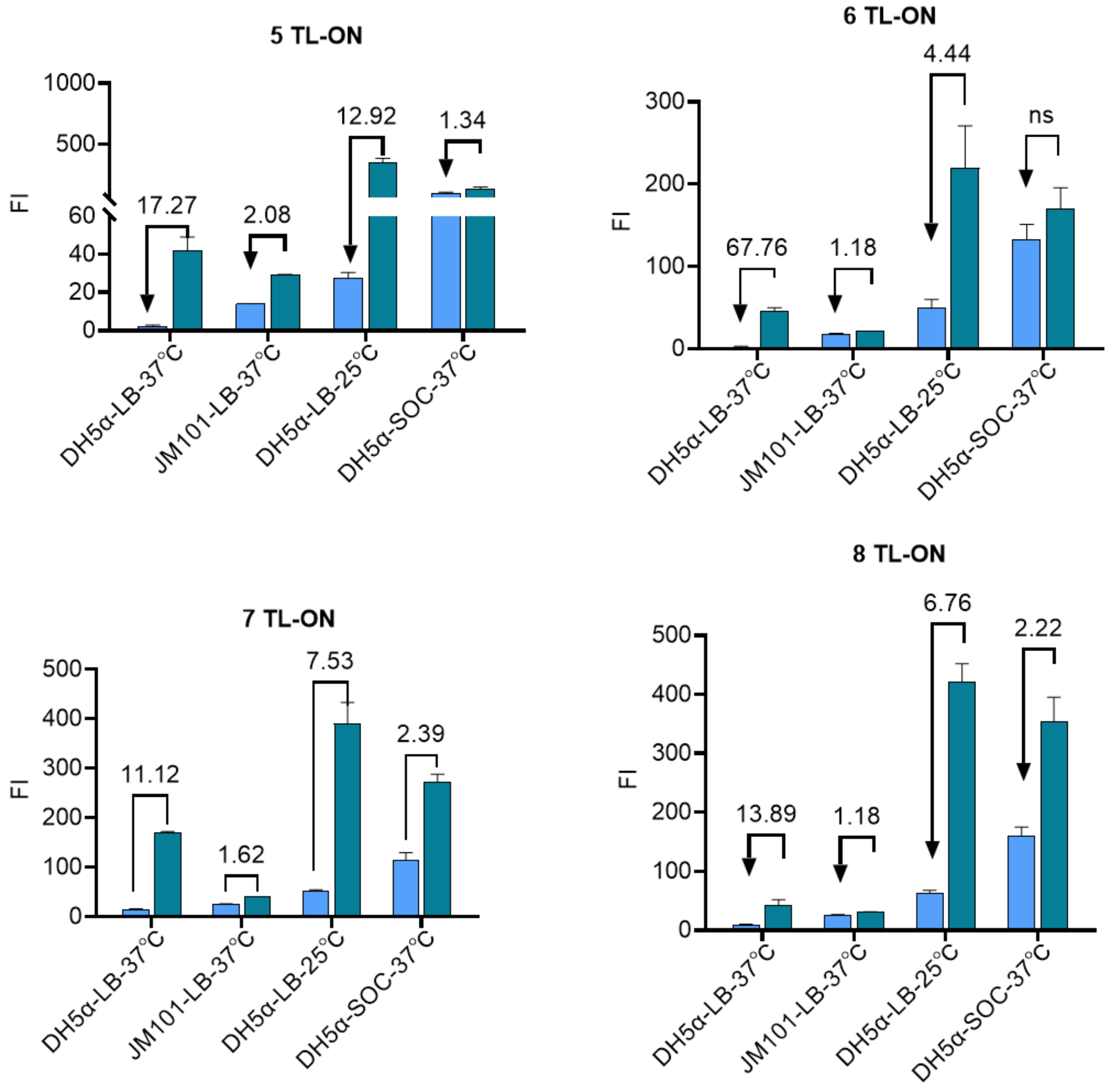
5 TL-ON
FI
1000
500
60
40
20
0
17.27
2.08
12.92
1.34
DH5α-LB-37°C
JM101-LB-37°C
DH5α-LB-25°C
DH5α-SOC-37°C
6 TL-ON
FI
300
200
100
0
4.44
ns
67.76
1.18
DH5α-LB-37°C
JM101-LB-37°C
DH5α-LB-25°C
DH5α-SOC-37°C
7 TL-ON
FI
500
400
300
200
100
0
7.53
2.39
11.12
1.62
DH5α-LB-37°C
JM101-LB-37°C
DH5α-LB-25°C
DH5α-SOC-37°C
8 TL-ON
FI
500
400
300
200
100
0
6.76
2.22
13.89
1.18
DH5α-LB-37°C
JM101-LB-37°C
DH5α-LB-25°C
DH5α-SOC-37°C

9 TL-ON

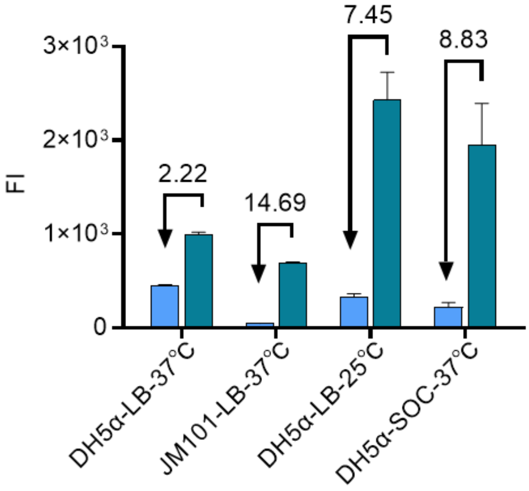

10 TL-ON

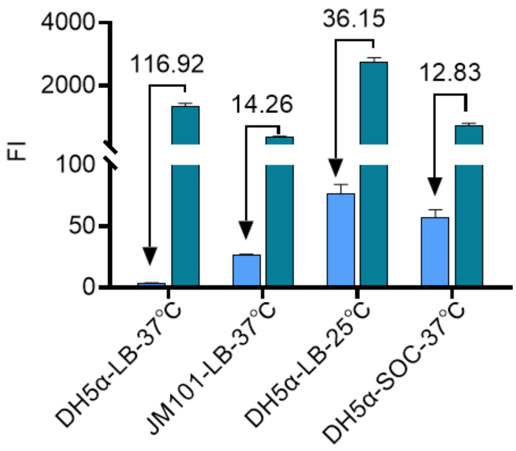

11 TL-ON

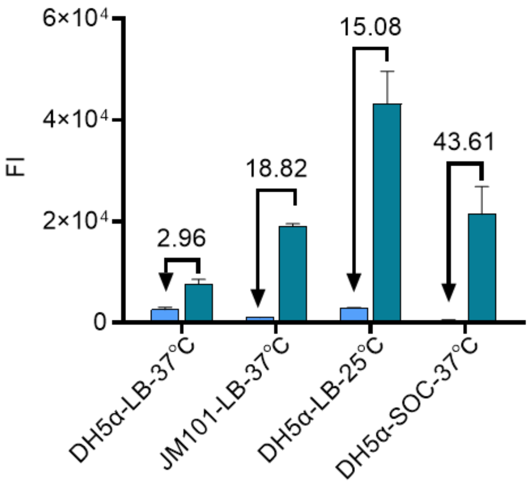

12 TL-ON

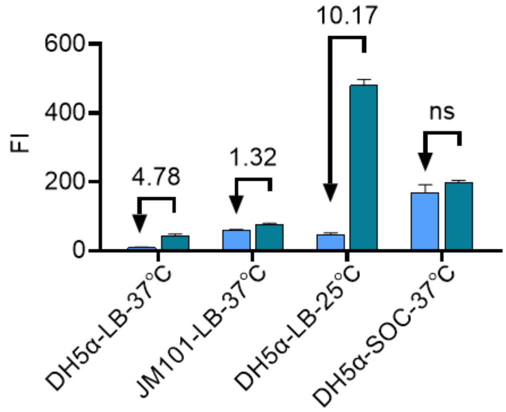

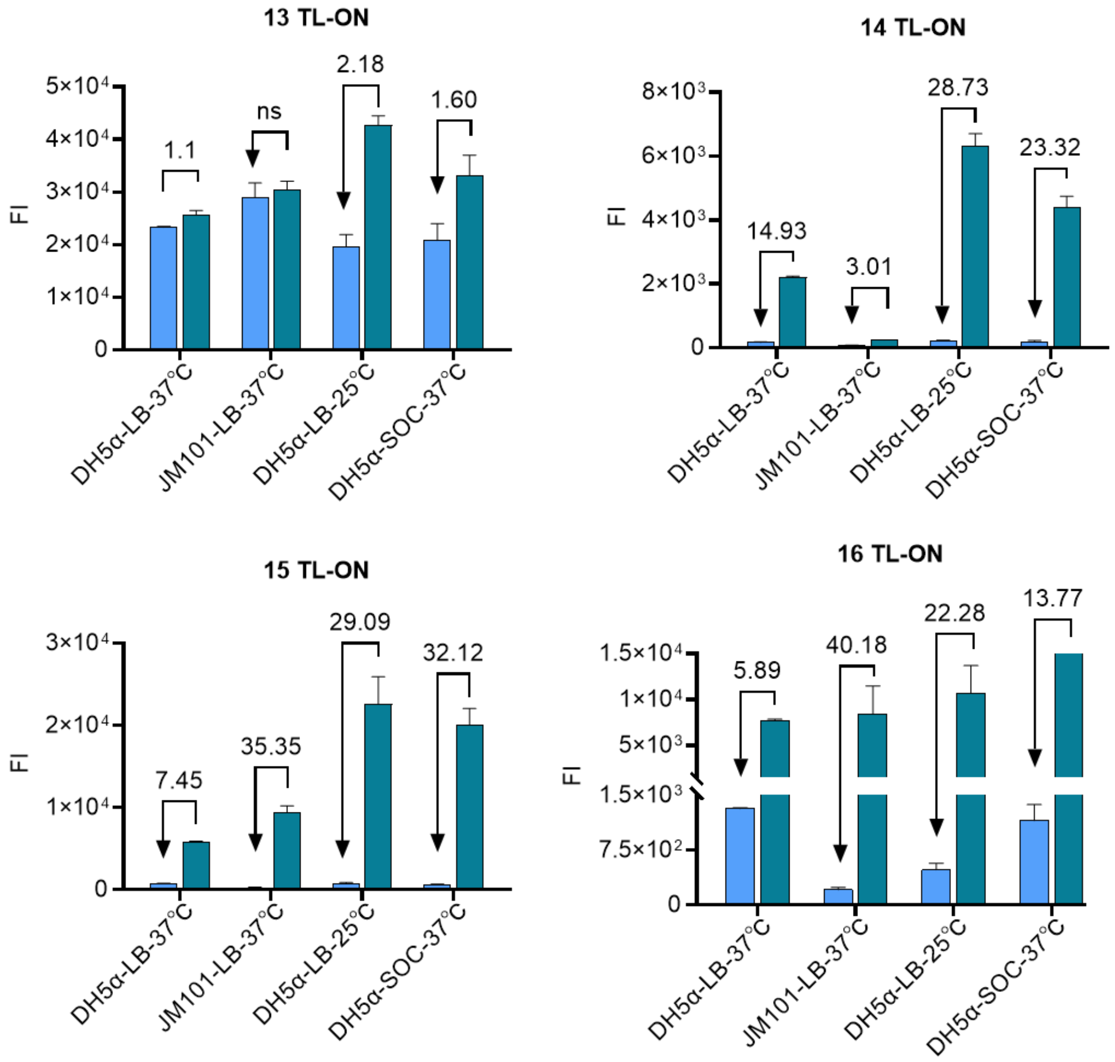
13 TL-ON
14 TL-ON
15 TL-ON
16 TL-ON
FI
1.1
ns
2.18
1.60
14.93
3.01
28.73
23.32
7.45
35.35
29.09
32.12
5.89
40.18
22.28
13.77
DH5α-LB-37°C
JM101-LB-37°C
DH5α-LB-25°C
DH5α-SOC-37°C

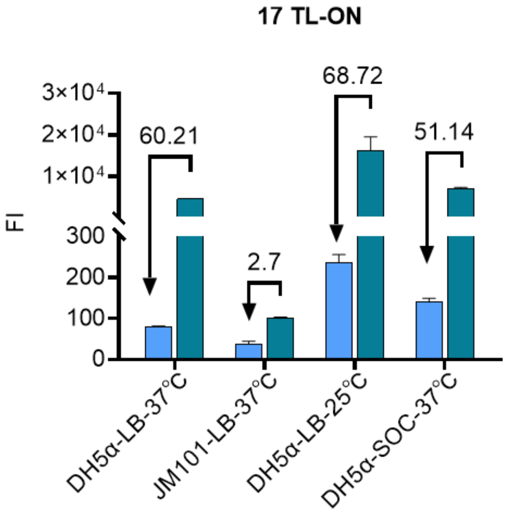
17 TL-ON
FI
3×10⁴
2×10⁴
1×10⁴
300
200
100
0
60.21
2.7
68.72
51.14
DH5α-LB-37°C
JM101-LB-37°C
DH5α-LB-25°C
DH5α-SOC-37°C

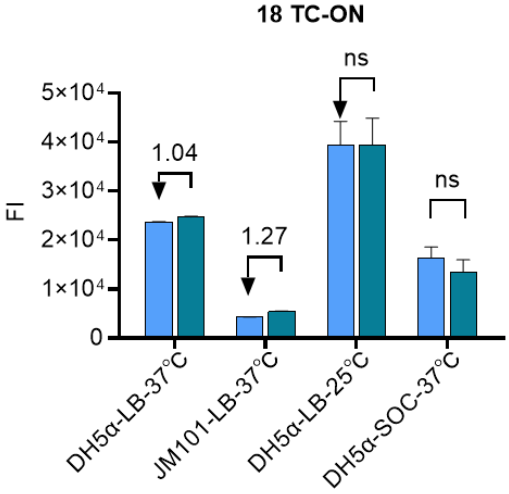
18 TC-ON
FI
5×10⁴
4×10⁴
3×10⁴
2×10⁴
1×10⁴
0
1.04
1.27
ns
ns
DH5α-LB-37°C
JM101-LB-37°C
DH5α-LB-25°C
DH5α-SOC-37°C

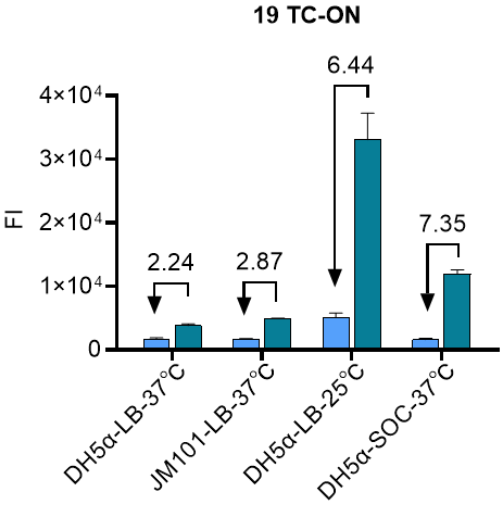
19 TC-ON
FI
4×10⁴
3×10⁴
2×10⁴
1×10⁴
0
2.24
2.87
6.44
7.35
DH5α-LB-37°C
JM101-LB-37°C
DH5α-LB-25°C
DH5α-SOC-37°C

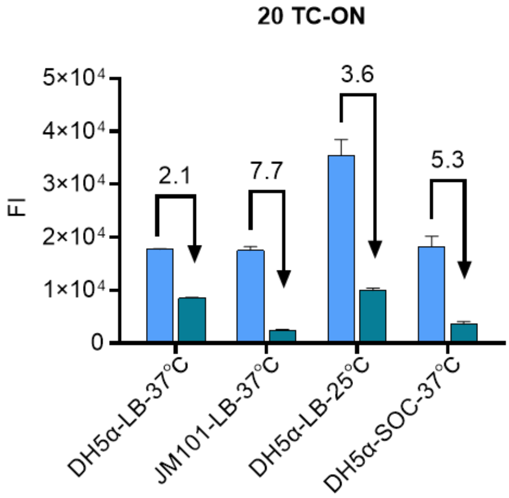
20 TC-ON
FI
5×10⁴
4×10⁴
3×10⁴
2×10⁴
1×10⁴
0
2.1
7.7
3.6
5.3
DH5α-LB-37°C
JM101-LB-37°C
DH5α-LB-25°C
DH5α-SOC-37°C

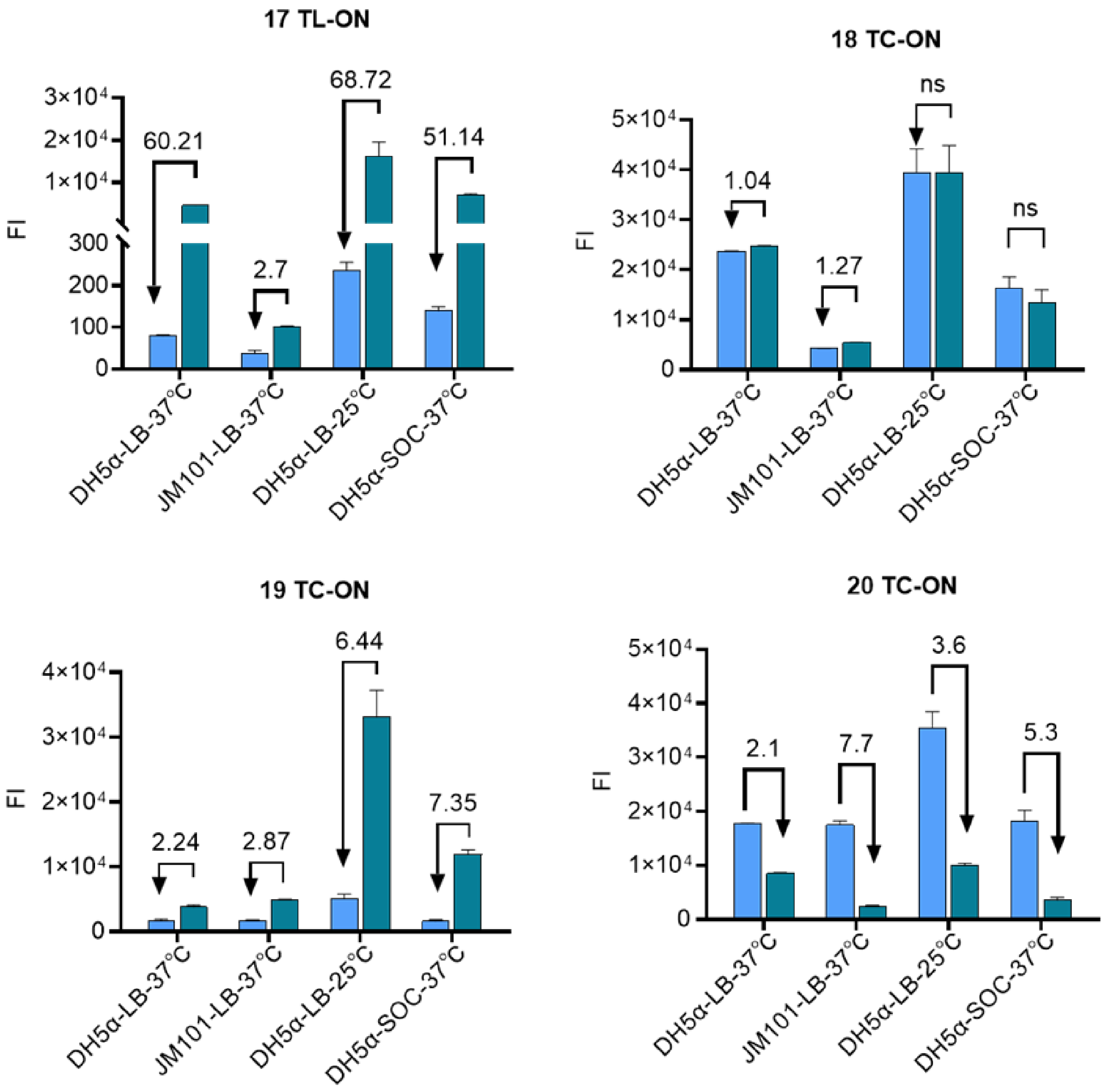
17 TL-ON
FI
3×10⁴
2×10⁴
1×10⁴
300
200
100
0
60.21
2.7
68.72
51.14
DH5α-LB-37°C
JM101-LB-37°C
DH5α-LB-25°C
DH5α-SOC-37°C
18 TC-ON
FI
5×10⁴
4×10⁴
3×10⁴
2×10⁴
1×10⁴
0
1.04
1.27
ns
ns
DH5α-LB-37°C
JM101-LB-37°C
DH5α-LB-25°C
DH5α-SOC-37°C
19 TC-ON
FI
4×10⁴
3×10⁴
2×10⁴
1×10⁴
0
2.24
2.87
6.44
7.35
DH5α-LB-37°C
JM101-LB-37°C
DH5α-LB-25°C
DH5α-SOC-37°C
20 TC-ON
FI
5×10⁴
4×10⁴
3×10⁴
2×10⁴
1×10⁴
0
2.1
7.7
3.6
5.3
DH5α-LB-37°C
JM101-LB-37°C
DH5α-LB-25°C
DH5α-SOC-37°C

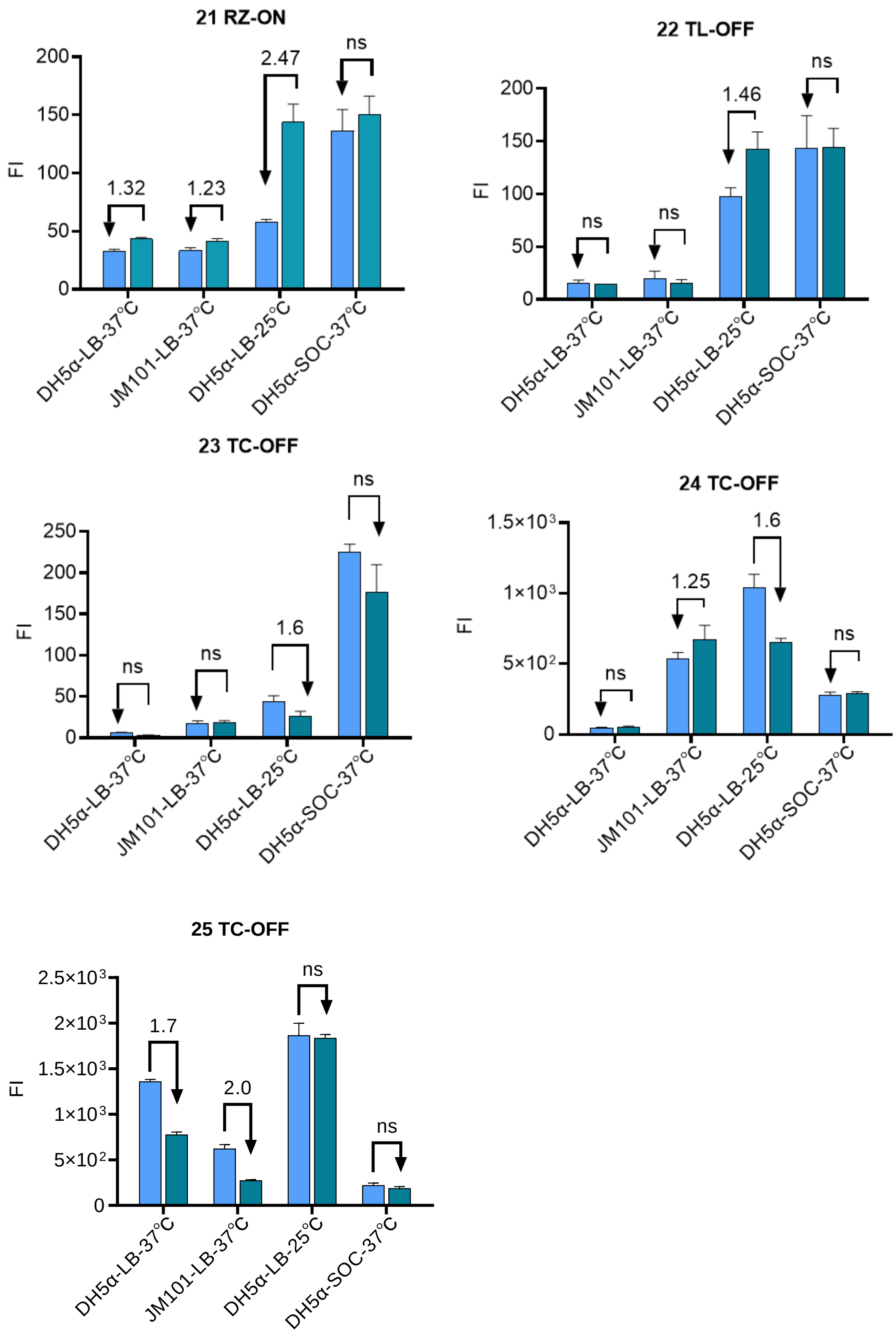

Supplementary Figure S2. Measurement of the regulatory effect of various theophylline riboswitches under different culture conditions.

Expression levels of TurboRFP FI were measured in the absence (light blue) and presence (dark blue) of 2 mM theophylline. The numbers above the column represent activation/repression ratios. Data represent mean ± SD of 3 biological replicates.

Supplementary Figure S3

pWA143 (No riboswitch)

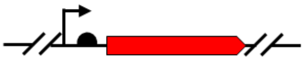

TGTGAGCGGATAACATTGACGGCTAGCTCAGTCCTAGGTACAGTGACGTCATCGATGGAGGAATTTTCC
ATGGGAAG

pWA131 (R1)

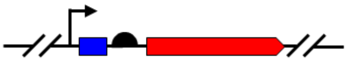

TGTGAGCGGATAACATTGACGGCTAGCTCAGTCCTAGGTACAGTGACGTCGGTGATACCAGCATCGTCT
TGATGCCCTTGGCAGCACCCGCTGCGCAGGGGGTATCAACCCCCTTTTTTTTTATCGATGGAGGAATTT
TCCATGGGAAG

pWA140 (R2)

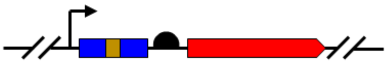

TGTGAGCGGATAACATTGACGGCTAGCTCAGTCCTAGGTACAGTGACGTCGGTGATACCAGCATCGTCT
TGATGCCCTTGGCAGCACCCGCTGCGCAGGGGGTATCAACCCCCTTTTTTTTTGTCTCAAAAAGAAGGT
GATACCAGCATCGTCTTGATGCCCTTGGCAGCACCCGCTGCGCAGGGGGTATCAACCCCCTTTTTTTTT
GGAGGAATTTTCCATGGGAAG

pWA141 (R3)

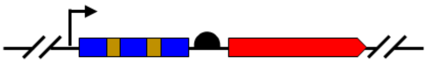

TGTGAGCGGATAACATTGACGGCTAGCTCAGTCCTAGGTACAGTGACGTCGGTGATACCAGCATCGTCT
TGATGCCCTTGGCAGCACCCGCTGCGCAGGGGGTATCAACCCCCTTTTTTTTTGTCTCAAAAAGAAGGT
GATACCAGCATCGTCTTGATGCCCTTGGCAGCACCCGCTGCGCAGGGGGTATCAACCCCCTTTTTTTTT
GTCTCAAAAAGAAGGTGATACCAGCATCGTCTTGATGCCCTTGGCAGCACCCGCTGCGCAGGGGGTAT
CAACCCCCTTTTTTTTTGGAGGAATTTTCCATGGGAAG

pWA144 (RepA)

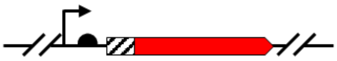

TGTGAGCGGATAACATTGACGGCTAGCTCAGTCCTAGGTACAGTGACGTCAATTTTAGAAAGTCTAGAG
GAGGAATTTTCCATGATGAACCAGAGCTTTATTAGCGATATTCTGTATGCGGATATTGAATCCATGGGAA
G

pWA146 (R2-RepA)

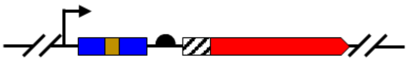

TGTGAGCGGATAACATTGACGGCTAGCTCAGTCCTAGGTACAGTGACGTCGGTGATACCAGCATCGTCT
TGATGCCCTTGGCAGCACCCGCTGCGCAGGGGGTATCAACCCCCTTTTTTTTTGTCTCAAAAAGAAGGT
GATACCAGCATCGTCTTGATGCCCTTGGCAGCACCCGCTGCGCAGGGGGTATCAACCCCCTTTTTTTTT
GGAGGAATTTTCCATGAACCAGAGCTTTATTAGCGATATTCTGTATGCGGATATTGAATCCATGGGAAG

Supplementary Figure S3. DNA Sequences upstream of *turboRFP*.

The -35 and -10 regions of the promote J23100 are indicated by blue letters. The translation start codon and RBS of *turborfp* are indicated by red letters. The theophylline riboswitch coding sequences are shown in blue background. The linker coding sequences are shown in dark brown background. The RepA-tag coding sequences are shown in dark green background.

Supplementary Figure S4

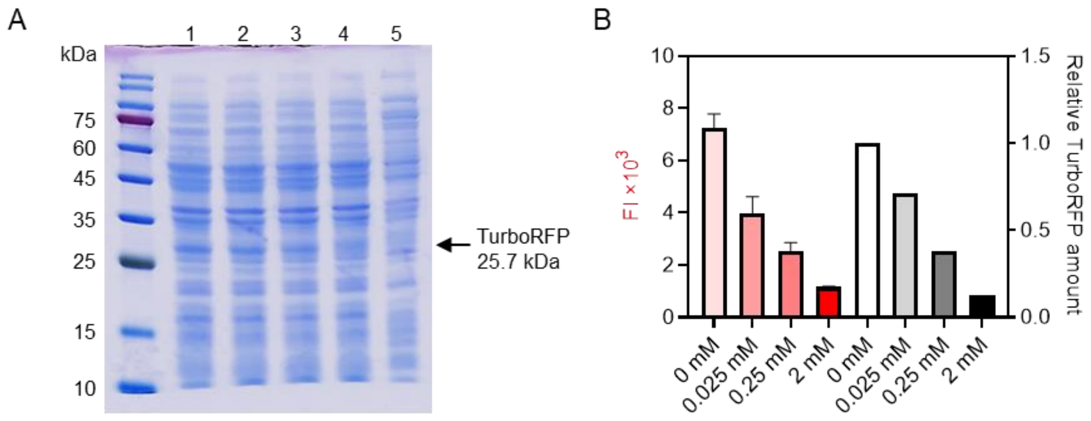

Supplementary Figure S4. The relationship between TurboRFP amount and its FI.

(A) SDS-PAGE gel showing *E. coli* total protein profile. Lane 1-4: MG1655-pWA140 grown in LB medium supplemented with 0, 0.025, 0.25 and 2 mM theophylline and 100 µg/mL ampicillin; Lane 5, MG1655-pBRPcon, grown in LB medium supplemented with 100 µg/mL ampicillin. (B) The TurboRFP FI of MG1655-pWA140 grown in LB medium supplemented with 0, 0.025, 0.25 and 2 mM theophylline and 100 µg/mL ampicillin were shown as red bars. The band intensities of TurboRFP in (A) were quantified by Image J and their expressions were normalized to the total protein amount, and the relative expression of TurboRFP was shown as grey bars.

Supplementary Table S1. A complete list of theophylline riboswitches published to date**.**

**Number**	**Sequence***	**Length**	**Regulation mechanism**	**Name in the literature**	**Strain**	**Growth condition**	**Construction method**	**Activation/repression ratio**	**Reference**
1	UAUGUUGAUACU UAAUUUAAAGAU UAAACAAAAGAU GAUACCAGCCGA AAGGCCCUUGGC AGCUCUCGUGGA GUGGAUGAAGUG	84 bp	Translational ON	mTCT-8	*B. subtilis* WH335	*Bacillus* strains were grown in minimal medium supplemented with 0.5% succinate at 37 ℃ and harvested at an A600 =0.4 and 0.8, respectively. β-galactosidase activity expressed from the *xylA*::*lacZ* fusion was measured in the absence and presence of 0.2% xylose and 10 mM theophylline.	We then replaced the stem loop by a rationally designed RNA switch which is composed of the theophylline aptamer as receptor domain and a communication domain class II-1 inhibition element	Indirect measurements: the repression ratio of *xylA*::*lacZ* fusion was about 4-fold. However, the riboswitch regulates XylR, *xylA*::*lacZ* fusion was negatively regulated by XylR. Repression ratio of XylR was not measured.	(3)
2	CCCGGUACCGGU GAUACCAGCAUC GUCUUGAUGCCC UUGGCAGCACCU AUAAAGACAACA AGAUGUGCGAAC UCG	75 bp	Translational ON	mTCT-8	*E. coli* TOP10	Cells were grown in LB-media supplemented with ampicillin (50 μg/mL) and 500 μM theophylline at 37 ℃ with shaking to an OD_{600} of 0.3-0.5, and β-galactosidase activity was assayed spectrophotometrically by monitoring the hydrolysis of o-nitrophenyl-β-D-glucopyranoside.	We subcloned the mTCT8-4 aptamer sequence at a location five bp upstream of the ribosome binding site of the β-galactosidase reporter gene (IS10-*lacZ*) in the plasmid pLacZU1hpII.	A signal to background ratio (activation ratio) of approximately eight in the presence of 500 μM theophylline.	(4)

3	GGUGAUACCAGCAUCGUCUUGAUGCCCUUGGCAGCACCCCGCUGCAGGACAACAAGAUG	59 bp	Translational ON	8.1*	*E. coli* TOP10	*Bacillus* strains were grown in minimal medium supplemented with 0.5% succinate at 37 ℃ and harvested at an A600 =0.4 and 0.8, respectively. β-galactosidase activity expressed from the *xylA*::*lacZ* fusion was measured in the absence and presence of 0.2% xylose and 10 mM theophylline.	We then replaced the stem loop by a rationally designed RNA switch which is composed of the theophylline aptamer as receptor domain and a communication domain class II-1 inhibition element	Indirect measurements: the repression ratio of *xylA*::*lacZ* fusion was about 4-fold. However, the riboswitch regulates XylR, *xylA*::*lacZ* fusion was negatively regulated by XylR. Repression ratio of XylR was not measured.	(5)
4	GGUGAUACCAGCAUCGUCUUGAUGCCCUUGGCAGCACCCUGCUAAGGUAACAACAAGAUG	60 bp		12.1		Cells were grown in LB-media supplemented with ampicillin (50 μg/mL) and 500 μM theophylline at 37 ℃ with shaking to an OD_{600} of 0.3-0.5, and β-galactosidase activity was assayed spectrophotometrically by monitoring the hydrolysis of o-nitrophenyl-β-D-galactopyranoside.	We subcloned the mTCT8-4 aptamer sequence at a location five bp upstream of the ribosome binding site of the β-galactosidase reporter gene (IS10-*lacZ*) in the plasmid pLacZU1hpII.	A signal to background ratio (activation ratio) of approximately eight in the presence of 500 μM theophylline.	
5	GGUACCGGUGAUACCAGCAUCGUCUUGAUGCCCUUGGCAGCACCCUGAGAAGGGGCAACAAGAUG	65 bp	Translational ON	Riboswich A	Various bacterial strains	Electrocompetent *E. coli* cells (strain MDS42, Scarab Genomics) were prepared and transformed with the pBAV1K riboswitch constructs by electroporation.	The promoter and constant 5'-UTR sequence (described in the text of Table S1) is positioned between *Xba*I and *Kpn*I sites; the riboswitch sequence (Table S1) is	Displayed more than 25-fold activation ratio.	

6	GGUACCGGUGAU<br>ACCAGCAUCGUC<br>UUGAUGCCCUUG<br>GCAGCACCCGCU<br>GCGCAGGGGGUA<br>UCAACAAGAUG	71 bp		Riboswitch B		All β-galactosidase assays were performed by the method of Miller, using cells that were grown with shaking at 37 ℃ in Luria Broth (LB) containing kanamycin (50 μg/mL) and 0 or 2 mM theophylline.	positioned between the *Kpn*I site and the start codon; and the stop codon of the reporter gene (IS10-*lacZ*, in this case) precedes the *Spe*I recognition site.		(6)
7	GGUACCUGAUAA<br>GAUAGGGGUGAU<br>ACCAGCAUCGUC<br>UUGAUGCCCUUG<br>GCAGCACCAAGA<br>CAACAAGAUG	70 bp		Riboswitch C					
8	GGUACCGGUGAU<br>ACCAGCAUCGUC<br>UUGAUGCCCUUG<br>GCAGCACCCUGC<br>UAAGGUAACAAC<br>AAGAUG	66 bp		Riboswitch D					
9	GGUACCGGUGAU<br>ACCAGCAUCGUC<br>UUGAUGCCCUUG<br>GCAGCACCCUGC<br>UAAGGAGGUAAC<br>AACAAGAUG	69 bp		Riboswitch E					
10	GGUACCGGUGAU<br>ACCAGCAUCGUC<br>UUGAUGCCCUUG<br>GCAGCACCCUGC<br>UAAGGAGGCAAC<br>AAGAUG	66 bp		Riboswitch E*					

11	AUACGACUCACU AUAGGUGAUACC AGCAUCGUCUUG AUGCCCUUGGCA GCACCCUGCUAA AGGAGGUAACAA CAAGAUG	79 bp	Translational ON	Riboswitch E1	*B. subtilis*	All of the strains were cultured with aeration in Luria–Bertani (LB) at 37 ℃. When appropriate, the *B. subtilis* growth medium was supplemented with kanamycin (5 µg/mL), and the *E. coli* growth medium was supplemented with ampicillin (10 µg/mL). GFP expression level increased linearly from 0 to 4 mM and continued to increase for the higher concentration of theophylline (up to 8 mM).	A strong constitutive promoter, P43, was placed upstream of the synthetic riboswitch E1 (derived from the reported riboswitch E). By using green fluorescent protein (GFP) as a reporter gene, riboswitch E1 with P43 was able to achieve inducible expression in *B. subtilis*.	The induction ratios constantly increased during the induction period, and were consistent with the expression levels. Expression due to leakage remained low during the induction period. Finally, the induction ratio peaked at 4.3 after 24 h of induction	(7)
12	AACGGGACUCAC UAUAGGUACCGG UGAUACCAGCAU CGUCUUGAUGCC CUUGGCAGCACC CUGCGGGCCGGG CAACAAGAUG	82 bp	Translational ON		*E. coli* DH10B	Cells harboring cloned plasmids expressing the mRFP1 reporter were grown overnight in 700 µl LB media and 50 µg/ml Cm antibiotic in a 96 deep-well plate at 37 ℃ and 200 rpm orbital shaking. Cells were then diluted to 0.01 into fresh media, and grown until reaching mid-exponential phase. To characterize their ON states, added ligand concentrations were 2 mM theophylline.	Theophylline riboswitches were constructed and inserted into an mRFP1 fluorescent protein expression vector, derived from plasmid pFTV1 (ColE1 origin, Cm^R). Three theophylline riboswitches (Theo-40, Theo-41 and Theo-45) also controlled the translation of a fusion mRFP1 protein. To create the fusion protein, four or five non-rare codons were introduced between the start codon and *Sac*I restriction	Riboswitches have ARmax<3.0 (Riboswitch Specific activation ratio) when they bind theophylline (2 mM)	(8)

							site within mRFP1 coding section. All the riboswitches were constructed using standard molecular cloning.		
13	CACUGUUCGUCA AGAAAGCAUCAU UGUGACUGUGUA GAUUGCUAUUAC AAGAAGAUCAGG AGCAAACUAUG	71 bp	Translational ON	Hit 3-5	*E. coli*	Strains were grown at 37 ℃ with orbital shaking at 250 rpm in LB broth or M9 minimal medium containing 0.8% w/v glycerol. Fluorescence assays were performed following rounds of selections and screens. Strains were incubated overnight in M9 medium. Cultures were diluted to an OD_{600} of 0.05 and incubated until they reached an OD_{600} of 0.2−0.3. The cultures were split into two fractions and incubated with or without theophylline (1 mM).	The N40 plasmid library consists of a degenerate 40-nucleotide sequence, the ThiM#2 expression platform, RBS, and tetA-gfp fusion.	Our best riboswitch (Hit 3−5) displays 2.3-fold activation of downstream gene expression in the presence of theophylline (1mM). Random mutagenesis of Hit 3−5, coupled with selections and screens, afforded improved riboswitches displaying nearly 3-fold activation.	(9)
14	GGUGAUACCAGC AUCGUCUUGAUG CCCUUGGCAGCA CCCUGCUAAGGA GGUAACAACAUG	60 bp	Translational ON	Riboswitch F	*Clostridium*	Media for *clostridial* strains were supplemented with the following antibiotic/inducer/supplement when appropriate: thiamphenicol (15 μg/mL), erythromycin (10 μg/mL), cefotoxin (16 μg/mL), d-cycloserine (500 μg/mL), theophylline (0.1–10 mM),	The different riboswitches and the reporter *catP* were assembled using the type IIS restriction endonuclease *Bsa*I and introduced into the application-specific module *via Not*I and *Xho*I restriction sites. To prevent transcriptional read-through, this vector includes the	In the presence of 2 mM theophylline, the activation ratio was about 12.5-fold.	(10)
15	GGUGAUACCAGC AUCGUCUUGAUG CCCUUGGCAGCA CCCUGCUAAGGA GGUAACUUAAUG	60 bp		Riboswitch G				In the presence of 2 mM theophylline, the activation ratio was about 16-fold.	

16	GGUGAUACCAGCAUCGUCUUGAUGCCCUUGGCAGCACCCUGCUAAGGAGGUGUGUUAAUG	60 bp		Riboswitch H		glucose 0.05% w/v. *Clostridium* strains were grown at 37 ℃ in an anaerobic cabinet (MG1000 anaerobic workstation; Don Whitley Scientific Ltd.).	terminator Tfdx downstream of the reporter.	In the presence of 2 mM theophylline, the activation ratio was about 4.9-fold.	
17	GGUGAUACCAGCAUCGUCUUGAUGCCCUUGGCAGCACCCUGCUAAGGAGGUCAACAAGAUG	61 bp		Riboswitch I				In the presence of 2 mM theophylline, the activation ratio was about 12-fold.	
18	CAGGUGAUACCAGCAUCGUCUUGAUGCCCUUGGCAGCACCTATATAAGAAGAAGGGUACCUUAAACCCCUUCUUCUUAUGAAGAAGGGGUUUUUAUUUUGGAGGAAUUUUCCAUG	99 bp	Transcriptional ON	mTCT-8	*E. coli* DH5a	All cells were grown in LB broth supplemented with ampicillin (50 μg/mL). Unless otherwise stated, the concentration of theophylline used for all experiments was 2 mM.	The *metI* transcriptional terminator (stem-loop followed by a polyU tract) was cloned in *Pst*I/*Pac*I with the *Kpn*I site added in to allow the aptamer (Y-shape) plus linker element to be cloned in *Pst*I/*Kpn*I.	The clones examined showed a 2- to 4-fold increase in GFP expression in response to theophylline.	(11)
19	AAGUGAUACCAGCAUCGUCUUGAUGCCCUUGGCAGCACUUCAGAAAUCUCUGAAGUGCUGUUUUUUUUAGGAGGUUAAUGAUG	83 bp	Transcriptional ON	RS10	*E. coli* TOP10	*E. coli* Top10 cells were grown in the presence or absence of 2 mM theophylline until OD_{600} of 0.5–0.6 in 25 ml LB medium supplied with ampicillin and arabinose.	Riboswitch constructs were generated by overlap extension polymerase chain reaction and fused to *bgaB* reporter in pBAD2_bgaB by Quick Change site-directed mutagenesis (Stratagene).	Three of the constructs turned out to stimulate reporter gene expression by 3-fold (RS10).	(12)

20	AAUUAAAUAGCU AUUAUCACGAUU UUAUACCAGCUU CGAAAGAAGCCC UUGGCAGAAAAU CCUGAUUACAAA AUUUGUUUAUGA CAUUUUUUGUAA UCAGGAUUUUUU UUGGAGGAATTTT CCAUG	143 bp	Transcriptional ON	theo/pbuE*	*E. coli* BW25113 (Dnep)	The culture was allowed to grow to early exponential phase (OD_{600}= 0.1–0.5) in CSB rich defined media. Ligand was added at varying concentration to the media at the concentrations indicated and the cells were allowed to grow for 6 (pbuE chimeras) h at 37 ℃.	Chimeric riboswitches were cloned upstream of a gfp reporter gene under control of a strong tac promoter into pRR1. *E. coli* strain BW25113 (Δ*nep*) [Keio collection] was transformed with the resultant vectors.	No *in vivo* data presented in the literature	(13)
21	GGGAGACCACAA CGGUUUCCCUAU CACCUUUUUGUA GGUUGCCCGAAA GGGCGACCCUGA UGAGCCUGGAUA CCAGCCGAAAGG CCCUUGGCAGUU AGACGAAACAAG AAGGAGAUAUAC CAAUG	125 bp	Ribozyme ON	mTCT-8	*E. coli* BL21 (DE3)	The cells were grown in LB at 37 ℃ until OD_{600} reached 0.5. 0–10 mM theophylline was added to it on ice. The mixture was incubated at 37 ℃ for 1.5–2.5 h until OD_{600} reached 0.8.	A plasmid that encodes a theophylline-induced aptazyme-based riboswitch sensor (TARS) under the control of the T7 promoter; in this sensor, the reporter gene is luciferase.	The ratio of luciferase activity in the presence of theophylline relative to its activity in the absence of theophylline was less than 1.5.	(14)

22	AUAGGUACCUAAUGCAACCUGAUACCAGCAUCGUCUUGAUGCCCUUGGCAGCAGGCAACAAG	62 bp	Translational OFF	mTCT-8	*E. coli* TOP10	LB media supplemented with ampicillin (50 mg/ml). Half-maximal repression is observed at an extra cellular theophylline concentration of 300 μM.	First, the RBS was incorporated within the aptamer structure in a region that is expected to be paired when theophylline is bound. The second modification to our previous libraries involved positioning the 8-nucleotide randomized region immediately to the aptamer. Finally, we replaced the start codon following the aptamer with TAT.	The most significant improvement arising from one of the parent five-fold repressors was for a specific NNY sequence (TAT) that now enabled 27-fold repression of gene expression in *E. coli*.	(15
23	AAUUUCAUAGUUAGAUCGUGUUAUAUGGUGAAGAUAAUACCAGCUUCGAAAGAAGCCCUUGGCAGUAUCUCGUUGUUCAUAAUCAUUUAUGAUGAUUAAUUGAUAAGCAAUGAGAGUAUUCCUCUCAUUGCUUUUUUUAUUGUGGACAAAGCGCUCUUUCUCCUCACCCGCACGAACCAAAAUGUAAAGGGUGGUAAUACAUG	138 bp	Transcriptional OFF	theo/lysC	*E. coli* BW25113 (Dnep)	Theophylline was added to the media at the concentrations from 0 to 3 mM. The cells were allowed to grow for 6 h at 37 ℃. At this point 300 μL of the cultures was used to measure the OD_{600} and their FI in a plate reader.	A reporter plasmid for testing riboswitch function in *E. coli* with a *gfpuv* reporter gene was constructed using pBR322 as the parental vector using standard molecular biological techniques. The sequence of the chimeric riboswitch insert for *theo/metE* reporters are identical to those used for *in vitro* transcription assays. The resultant vectors were transformed into *E. coli* strain BW25113-(Δnep).	Chimeric riboswitch displays strong regulatory activity in *E. coli* as indicated by reduction in fluorescence to a background level upon addition of 1 mM effector to a defined medium. Figure 5d showed a repression fold of about 4 at 2 mM theophylline.	(16)

24	CUUCCUGACACGA<br>AAAUUUCAUAUCC<br>GUUCUUAAUACCA<br>GCUUCGAAAGAAG<br>CCCUUGGCAGUAA<br>GAAGAGACAAAAU<br>CACUGACAAAGUC<br>UUCUUCUUAAGAG<br>GACUUUUUUUAUU<br>UCUCUUUUUCCU<br>UGCUGAUGUGAAU<br>AAAGGAGGCAGAC<br>AAUG	114 bp	Transcriptional OFF	theo/yitJ					
/25	CAAAAAAUUAAUA<br>ACAUUUUCUCUUA<br>UACCAGCUUCGAA<br>AGAAGCCCUUGGC<br>AGGAGAGAGGCAG<br>UGUUUUACGUAGA<br>AAAGCCUCUUUCU<br>CUCAUGGGAAAGA<br>GGCUUUUUGUUGU<br>GAGAAAACCUCUU<br>AGCAGCCUGUAUC<br>CGCGGGUGAAAGA<br>GAGUGUUUUACAU<br>AUAAAGGAGGAGA<br>AACAAUG	112 bp	Transcriptional OFF	theo/metE					

Theophylline aptamers are marked in green. The translation start codon and RBS are marked in red. Ribozyme cleavage sites are marked in blue. Intrinsic terminators are marked in purple while the hairpin sequences are underlined.

Supplementary Table S2. Plasmids used in this study.

Plasmid	Description	Resources
pBRPlac	Amp^R, pBR322-derived plasmid	(17)
pBRPcon	Amp^R, pBRPlac-derived plasmid with its *lac* promoter replaced by the strong constitutive promoter J23100	This study
pWA131	Amp^R, pBRPcon derivative containing single theophylline riboswitch and *turborfp* coding sequence	This study
pWA140	Amp^R, pBRPcon derivative containing double tandem theophylline riboswitch coding sequence and *turborfp*	This study
pWA141	Amp^R, pBRPcon derivative containing triple tandem theophylline riboswitch coding sequence and *turborfp*	This study
pWA143	Amp^R, pBRPcon derivative containing only *turborfp*	This study
pWA144	Amp^R, pBRPcon derivative containing RepA-tag encoding sequence and *turborfp*	This study
pWA146	Amp^R, pBRPcon derivative containing double tandem theophylline riboswitch and RepA-tag coding sequence and *turborfp*	This study
pSF-001	Amp^R, pBRPcon derivative containing No. 1 theophylline riboswitch coding sequence and *turborfp*	This study
pSF-002	Amp^R, pBRPcon derivative containing No. 2 theophylline riboswitch coding sequence and *turborfp*	This study
pSF-003	Amp^R, pBRPcon derivative containing No. 22 theophylline riboswitch coding sequence and *turborfp*	This study
pSF-004	Amp^R, pBRPcon derivative containing No. 21 theophylline riboswitch coding sequence and *turborfp*	This study
pSF-005	Amp^R, pBRPcon derivative containing No. 18 theophylline riboswitch coding sequence and *turborfp*	This study
pSF-006	Amp^R, pBRPcon derivative containing No. 3 theophylline riboswitch coding sequence and *turborfp*	This study
pSF-007	Amp^R, pBRPcon derivative containing No. 4 theophylline riboswitch coding sequence and *turborfp*	This study
pSF-008	Amp^R, pBRPcon derivative containing No. 5 theophylline riboswitch coding sequence and *turborfp*	This study
pSF-009	Amp^R, pBRPcon derivative containing No. 6 theophylline riboswitch coding sequence and *turborfp*	This study
pSF-010	Amp^R, pBRPcon derivative containing No. 7 theophylline riboswitch coding sequence and *turborfp*	This study
pSF-011	Amp^R, pBRPcon derivative containing No. 8 theophylline riboswitch coding sequence and *turborfp*	This study
pSF-012	Amp^R, pBRPcon derivative containing No. 9 theophylline riboswitch coding sequence and *turborfp*	This study
pSF-013	Amp^R, pBRPcon derivative containing No. 10 theophylline riboswitch coding sequence and *turborfp*	This study
pSF-014	Amp^R, pBRPcon derivative containing No. 19 theophylline riboswitch coding sequence and *turborfp*	This study
pSF-015	Amp^R, pBRPcon derivative containing No. 23 theophylline riboswitch coding sequence and *turborfp*	This study
pSF-016	Amp^R, pBRPcon derivative containing No. 24 theophylline riboswitch coding sequence and *turborfp*	This study
pSF-017	Amp^R, pBRPcon derivative containing No. 25 theophylline riboswitch coding sequence and *turborfp*	This study
pSF-018	Amp^R, pBRPcon derivative containing No. 20 theophylline riboswitch coding sequence and *turborfp*	This study
pSF-019	Amp^R, pBRPcon derivative containing No. 11 theophylline riboswitch coding sequence and *turborfp*	This study
pSF-020	Amp^R, pBRPcon derivative containing No. 12 theophylline riboswitch coding sequence and *turborfp*	This study
pSF-021	Amp^R, pBRPcon derivative containing No. 13 theophylline riboswitch coding sequence and *turborfp*	This study

pSF-022	Amp^R, pBRPcon derivative containing No. 14 theophylline riboswitch coding sequence and *turborfp*	This study
pSF-023	Amp^R, pBRPcon derivative containing No. 15 theophylline riboswitch coding sequence and *turborfp*	This study
pSF-024	Amp^R, pBRPcon derivative containing No. 16 theophylline riboswitch coding sequence and *turborfp*	This study
pSF-025	Amp^R, pBRPcon derivative containing No. 17 theophylline riboswitch coding sequence and *turborfp*	This study
pMV261	*Kan*^{R}, a *M. smegmatis* plasmid, pAL5000 replicon, colE1 replicon, hsp60 promoter	(18)
pMV262	*Kan*^{R}, pMV261 derivative containing R2 coding sequence and *turborfp*	This study
pRP0122	*Spec*^{R}, plasmid used in *B. thuringiensis*, *aadA*, pSC101 replicon, Pbe-*amcyan-Bc3-5-turborfp*	(19)
pRP2TRTR	*Spec*^{R}, pRP0122 derivative containing R2 coding sequence and *turborfp*	This study
pHT43	Amp^R, a *B. subtilis* plasmid, pUC replicon	(20)
pHT44	Amp^R, pHT43 derivative containing R2 coding sequence and *turborfp*	This study
pCas	*repA101*(Ts) *kan Pcas-cas9 ParaB-Red lacI*^{q} *Ptrc*-sgRNA-pMB1	(21)
pTargetF-pMB1	pMB1 *aadA sgRNA-pMB1*	(21)
pWA164	pTarget-*lacZ*-sgRNA	This study
pWA169	pTarget- *lacZ*-sgRNA with 400 bp upstream and downstream homologous arm and coding sequence of R2	This study
pWA170	pTarget- *lacZ*-sgRNA with 400 bp upstream and downstream homologous arm and coding sequence of RR	This study

Supplementary Table S3. Strains used in this study.

Strains	Description	Source
**Start strains**		
E. coli DH5α	F^- *endA1 glnV44 thi-1 recA1 relA1 gyrA96 deoR nupG purB20 φ80dlacZΔM15* Δ (*lacZYA-argF*) U169, hsdR17 ($rK^- mK^+$), λ^-	laboratory stocked
E. coli MG1655	K-12 F^- λ^- *ilvG*$^-$ *rfb-50 rph-1*	laboratory stocked
E. coli NST74	*aroH367*, *tyrR366*, *tna-2*, *lacY5*, *aroF394 (fbr) malT384*, *pheA101 (fbr)*, *pheO352*, *aroG397 (fbr)*	(22)
E. coli BL21	*E. coli* B F^- *ompT gal dcm lon hsdSB* ($rB^- mB^-$) *[malB+]* $_{K\text{-}12}$ *(λS)*	laboratory stocked
E. coli HB101	F^- *mcrB mrr hsdS20* ($rB^- mB^-$) *recA13 leuB6 ara-14 proA2 lacY1 galK2 xyl-5 mtl-1 rpsL20* (Sm^R) *glnV44* λ^-	laboratory stocked
E. coli JM101	*glnV44 thi-1* Δ (*lac-proAB*) F'[$lacI^q$*ZΔM15 traD36* $proAB^+$]	laboratory stocked
E. coli BW25113	$lacI^+ rrnB_{T14}$ $\Delta lacZ_{WJ16}$ *hsdR*514 $\Delta araBAD_{AH33}$ $\Delta rhaBAD_{LD78}$ *rph-1* Δ*(araB–D)567* Δ *(rhaD–B) 568* Δ*lacZ4787* (::*rrnB-3*) *hsdR514 rph-1*	laboratory stocked
E. coli Top10	F- *mcrA* Δ(*mrr-hsdRMS-mcrBC*) *φ80lacZ*Δ*M15* Δ*lacX74 nupG recA1 araD139* Δ(*ara-leu*) 7697 *galE15 galK16 rpsL* (Str^R) *endA1* λ^-	laboratory stocked
Salmonella enterica serovar Typhimurium SL1344	*hisG46 rpsL*; virulent strain	(23)
Mycobacteria smegmatis MC^2155	Wild-type *M. smegmatis* strain	(24)
B. thuringiensis BMB171	*B. thuringiensis* strain BMB171; an acrystalliferous mutant strain; high transformation frequency	(25)
B. subtilis 168	Wild-type *B. subtilis* strain 168	laboratory stocked
**Derivative strains**		
MG1655-pSF001	MG1655 containing plasmid pSF001	This study
MG1655-pSF002	MG1655 containing plasmid pSF002	This study
MG1655-pSF003	MG1655 containing plasmid pSF003	This study
MG1655-pSF004	MG1655 containing plasmid pSF004	This study
MG1655-pSF005	MG1655 containing plasmid pSF005	This study
MG1655-pSF006	MG1655 containing plasmid pSF006	This study
MG1655-pSF007	MG1655 containing plasmid pSF007	This study
MG1655-pSF008	MG1655 containing plasmid pSF008	This study
MG1655-pSF009	MG1655 containing plasmid pSF009	This study
MG1655-pSF010	MG1655 containing plasmid pSF010	This study
MG1655-pSF011	MG1655 containing plasmid pSF011	This study
MG1655-pSF012	MG1655 containing plasmid pSF012	This study

Strains	Description	Source
MG1655-pSF013	MG1655 containing plasmid pSF013	This study
MG1655-pSF014	MG1655 containing plasmid pSF014	This study
MG1655-pSF015	MG1655 containing plasmid pSF015	This study
MG1655-pSF016	MG1655 containing plasmid pSF016	This study
MG1655-pSF017	MG1655 containing plasmid pSF017	This study
MG1655-pSF018	MG1655 containing plasmid pSF018	This study
MG1655-pSF019	MG1655 containing plasmid pSF019	This study
MG1655-pSF020	MG1655 containing plasmid pSF020	This study
MG1655-pSF021	MG1655 containing plasmid pSF021	This study
MG1655-pSF022	MG1655 containing plasmid pSF022	This study
MG1655-pSF023	MG1655 containing plasmid pSF023	This study
MG1655-pSF024	MG1655 containing plasmid pSF024	This study
MG1655-pSF025	MG1655 containing plasmid pSF025	This study
MG1655-pWA131	MG1655 containing plasmid pWA131	This study
MG1655-pWA140	MG1655 containing plasmid pWA140	This study
MG1655-pWA141	MG1655 containing plasmid pWA141	This study
MG1655-pWA143	MG1655 containing plasmid pWA143	This study
MG1655-pWA144	MG1655 containing plasmid pWA144	This study
MG1655-pWA146	MG1655 containing plasmid pWA146	This study
ST-pWA140	*S.* typhimurium SL1344 containing plasmid pWA140	This study
MS-pMV262	*M. smegmatis* MC^2155 containing plasmid pMV262	This study
BT-pRP2TRTR	*B. thuringiensis* BMB171 containing plasmid pRP2TRTR	This study
BS-pHT44	*B. subtilis* 168 containing plasmid pHT44	This study
BL21-pWA140	BL21 containing plasmid pWA140	This study

Strains	Description	Source
JM101-pWA140	JM101 containing plasmid pWA140	This study
HB101-pWA140	HB101 containing plasmid pWA140	This study
NST74-pWA140	NST74 containing plasmid pWA140	This study
BW25113-pWA140	BW25113 containing plasmid pWA140	This study
Top10-pWA140	Top10 containing plasmid pWA140	This study
DH5α-pWA140	DH5α containing plasmid pWA140	This study
R2-*lacZ*	The 123 bp intergenic region between *lacI* and *lacZ* was replaced by R2	This study
RR-*lacZ*	The 123 bp intergenic region between *lacI* and *lacZ* was replaced by RR system	This study

Supplementary Table S4. Primers used in this study.

Primer name	Primer sequences (5‘ to 3')	Usage
TRTR-Fp	GGTGATACCAGCATCGTCTTGATGCCCTTGGCAGCACCCGCTGCGCAGGGGGTAT	Theophylline riboswitch sequence synthesis
TRTR-Rp	GGAAAATTCCTCCATCGATAAAAAAAAAGGGGGTTGATACCCCCTGCGCAGCGGG	
TurboRFP-Fp	ATCGATGGAGGAATTTTCCATGGGAAGTGAATTGATTAAAGA	*turborfp* clone
TurboRFP-Rp	TTATCTATGCCCTAATTTACTAGGTAAATC	
131-VF	GACGATGCTGGTATCACCGACGTCAGTATCTTGTTATCCGCTC	pWA131 plasmid construction
131-VR	GTAAATTAGGGCATAGATAAAAGCTTTAATGCGGTAGTTTAT	
140-IF	TTTTTGTCTCAAAAAGAAGGTGATACCAGCATCGTCTT	pWA140, pWA141 plasmid construction
140-Rec-LP	TTCTTTTTGAGACAAAAAAAAAGGGGGTTGATACC	
143-Fp	AATTTTAGAAAGTCTAGAGGAGGAATTTTCCATGGGAAGTG	pWA143 plasmid construction
143-VF	TCTAGACTTTCTAAAATTGACGTCAGTATCTTGTTATCCG	
144-VF	CATACAGAATATCGCTAATAAAGCTCTGGTTCATGGAAAATTCCTCCTCTAGACTTTCT	pWA144 plasmid construction
144-IF	ATTAGCGATATTCTGTATGCGGATATTGAATCCATGGGAAGTGAATTGATTAAAGAAA	
BBa_J23104-IF	TTGACAGCTAGCTCAGTCCTAGGTATTGTGCTAGCGGTGATACCAGCATCGTCTTGATG	pWA146 plasmid construction
2TRTR-RepA-Rp	GGATTCAATATCCGCATACAGAAT	
rrsB-for	CAGAATGCCACGGTGAATACGTT	16S rRNA qRT-PCR
rrsB-rev	CAACCCAACTCCCATGGTGTGA	
turborfp-qrt-Fp	ACCATATGAGGGAACGCAAAC	*turborfp* qRT-PCR
turborfp-qrt-Rp	ACGTTCCCATGTAAATCCTTCA	
1-1-F	TATGTTGATACTTAATTTAAAGATTAAACAAAAGATGATACCAGCCGAAAGGCCCTT	No. 1 theophylline riboswitch synthesis
1-1-R	TTCATCCACTCCACGAGAGCTGCCAAGGGCCTTTCGGCTGGTATC	
Cre-1-F	GATAACAAGATACTGACGTCTATGTTGATACTTAATTTAAAGATTAAACAAAAGATG	pSF-001 plasmid construction
Cre-1-R	TTAATCAATTCACTTCCCATTTCATCCACTCCACGAGAGCTG	
1-ZT-F	ATGGGAAGTGAATTGATTAAAGAAAATATGCATATG	
1-ZT-R	GACGTCAGTATCTTGTTATCCGCTCACAATG	
2-1-F	GGTGATACCAGCATCGTCTTGATGCCCTTGGCAGCACCTATAAAGAC	No. 2 theophylline riboswitch synthesis
2-1-R	CGAGTTCGCACATCTTGTTGTCTTTATAGGTGCTGCCAAGGGCATCAAG	
Cre-2-F	GATAACAAGATACTGACGTCGGTGATACCAGCATCGTCTTGATG	pSF-002 plasmid construction
Cre-2-R	TTAATCAATTCACTTCCCATCGAGTTCGCACATCTTGTTGTC	

2-ZT-F	ATGGGAAGTGAATTGATTAAAGAAAATATGCATATG	
2-ZT-R	GACGTCAGTATCTTGTTATCCGCTCACAATG	
3-1-F	ATAGGTACCTAATGCAACCTGATACCAGCATCGTCTTGATGCCC	No. 22 theophylline riboswitch synthesis
3-1-R	ATACTTGTTGCCTGCTGCCAAGGGCATCAAGACGATGCTGGTATCAGG	
Cre-3-F	GAGCGGATAACAAGATACTGACGTCATAGGTACCTAATGCAACCTGATAC	pSF-003 plasmid construction
Cre-3-R	TTAATCAATTCACTTCCATACTTGTTGCCTGCTGCCAAG	
3-ZT-F	TATGGAAGTGAATTGATTAAAGAAAATATGCATATG	
3-ZT-R	GACGTCAGTATCTTGTTATCCGCTCACAATG	
4-1-F	GGGAGACCACAACGGTTTCCCTATCACCTTTTTGTAGGTTGCCCG	No. 21 theophylline riboswitch synthesis
4-1-R	GGCTGGTATCCAGGCTCATCAGGGTCGCCCTTTCGGGCAACCTACAAAAAGGTGATAGG	
4-2-F	AAGGGCGACCCTGATGAGCCTGGATACCAGCCGAAAGGCCCTTGGCAGTTAGACGAAAC	
4-2-R	TGGTATATCTCCTTCTTGTTTCGTCTAACTGCCAAGGGCCTTTC	
Cre-4-F	GATAACAAGATACTGACGTCGGGAGACCACAACGGTTTCC	pSF-004 plasmid construction
Cre-4-R	TTAATCAATTCACTTCCCATTGGTATATCTCCTTCTTGTTTCGTCTAACTG	
4-ZT-F	ATGGGAAGTGAATTGATTAAAGAAAATATGCATATG	
4-ZT-R	GACGTCAGTATCTTGTTATCCGCTCACAATG	
5-1-F	CTTGATGCCCTTGGCAGCACCTATATAAGAAGAAGGGTACCTTAA	No. 18 theophylline riboswitch synthesis
5-1-R	ATAAGAAGAAGGGGTTTAAGGTACCCTTCTTCTTATATAGGTGCT	
5-2-F	CAGGTGATACCAGCATCGTCTTGATGCCCTTGGCAGCAC	
5-2-R	AAAATAAAAACCCCTTCTTCATAAGAAGAAGGGGTTTAAGGTACCC	
Cre-5-F	GATAACAAGATACTGACGTCCAGGTGATACCAGCATCGTC	pSF-005 plasmid construction
Cre-5-R	TTCCCATGGAAAATTCCTCCAAAATAAAAACCCCTTCTTCATAAGAAG	
5-ZT-F	GGAGGAATTTTCCATGGGAAGTG	
5-ZT-R	GACGTCAGTATCTTGTTATCCGCTCACAATG	
6-1-F	GGTGATACCAGCATCGTCTTGATGCCCTTGGCAGCACCC	No. 3 theophylline riboswitch synthesis
6-1-R	CTTGTTGTCCTGCAGCGGGGTGCTGCCAAGGGCATCAAG	
Cre-6-F	GATAACAAGATACTGACGTCGGTGATACCAGCATCGTCTT	pSF-006 plasmid construction
Cre-6-R	TTAATCAATTCACTTCCCATCTTGTTGTCCTGCAGCGGGG	
6-ZT-F	ATGGGAAGTGAATTGATTAAAGAAAATATGCATATG	
6-ZT-R	GACGTCAGTATCTTGTTATCCGCTCACAATG	
7-1-F	GGTGATACCAGCATCGTCTTGATGCCCTTGGCAGCACCC	No. 4 theophylline riboswitch synthesis

7-1-R	CTTGTTGTTACCTTAGCAGGGTGCTGCCAAGGGCATCAAGAC	
Cre-7-F	GATAACAAGATACTGACGTCGGTGATACCAGCATCGTCTTGATG	pSF-007 plasmid construction
Cre-7-R	CTTTAATCAATTCACTTCCCATCTTGTTGTTACCTTAGCAGGGTGC	
7-ZT-F	ATGGGAAGTGAATTGATTAAAGAAAATATGCATATG	
7-ZT-R	GACGTCAGTATCTTGTTATCCGCTCACAATG	
8-1-F	GGTACCGGTGATACCAGCATCGTCTTGATGCCCTTGGCAGCACCC	No. 5 theophylline riboswitch synthesis
8-1-R	CTTGTTGCCCCTTCTCAGGGTGCTGCCAAGGGCATCAAGACGATG	
Cre-8-F	GATAACAAGATACTGACGTCGGTACCGGTGATACCAGCAT	pSF-008 plasmid construction
Cre-8-R	TTAATCAATTCACTTCCCATCTTGTTGCCCCTTCTCAGG	
8-ZT-F	ATGGGAAGTGAATTGATTAAAGAAAATATGCATATG	
8-ZT-R	GACGTCAGTATCTTGTTATCCGCTCACAATG	
9-1-F	GGTACCGGTGATACCAGCATCGTCTTGATGCCCTTGGCAGCACCCGCTGC	No. 6 theophylline riboswitch synthesis
9-1-R	CTTGTTGATACCCCCTGCGCAGCGGGTGCTGCCAAGGGCATCAAGACG	
Cre-9-F	GATAACAAGATACTGACGTCGGTACCGGTGATACCAGCATCG	pSF-009 plasmid construction
Cre-9-R	TTAATCAATTCACTTCCCATCTTGTTGATACCCCCTGCGC	
9-ZT-F	ATGGGAAGTGAATTGATTAAAGAAAATATGCATATG	
9-ZT-R	GACGTCAGTATCTTGTTATCCGCTCACAATG	
10-1-F	GGTACCTGATAAGATAGGGGTGATACCAGCATCGTCTTGATGCCCTTGG	No. 7 theophylline riboswitch synthesis
10-1-R	CTTGTTGTCTTGGTGCTGCCAAGGGCATCAAGACGATGCTGGTATCAC	
Cre-10-F	GATAACAAGATACTGACGTCGGTACCTGATAAGATAGGGGTGATAC	pSF-010 plasmid construction
Cre-10-R	TTAATCAATTCACTTCCCATCTTGTTGTCTTGGTGCTGCC	
10-ZT-F	ATGGGAAGTGAATTGATTAAAGAAAATATGCATATG	
10-ZT-R	GACGTCAGTATCTTGTTATCCGCTCACAATG	
11-1-F	GGTACCGGTGATACCAGCATCGTCTTGATGCCCTTGGCAGCACCCTG	No. 8 theophylline riboswitch synthesis
11-1-R	CTTGTTGTTACCTTAGCAGGGTGCTGCCAAGGGCATCAAGACGATG	
Cre-11-F	GATAACAAGATACTGACGTCGGTACCGGTGATACCAGCATCG	pSF-011 plasmid construction
Cre-11-R	TTAATCAATTCACTTCCCATCTTGTTGTTACCTTAGCAGGGTGC	
11-ZT-F	ATGGGAAGTGAATTGATTAAAGAAAATATGCATATG	
11-ZT-R	GACGTCAGTATCTTGTTATCCGCTCACAATG	
12-1-F	GGTACCGGTGATACCAGCATCGTCTTGATGCCCTTGGCAGCACCCTGC	No. 9 theophylline riboswitch synthesis
12-1-R	CTTGTTGTTACCTCCTTAGCAGGGTGCTGCCAAGGGCATCAAGACGATG	

Cre-12-F	GATAACAAGATACTGACGTCGGTACCGGTGATACCAGCATCG	pSF-012 plasmid construction
Cre-12-R	TTAATCAATTCACTTCCCATCTTGTTGTTACCTCCTTAGCAGGG	
12-ZT-F	ATGGGAAGTGAATTGATTAAAGAAAATATGCATATG	
12-ZT-R	GACGTCAGTATCTTGTTATCCGCTCACAATG	
13-1-F	GGTACCGGTGATACCAGCATCGTCTTGATGCCCTTGGCAGCACCCTG	No.10 theophylline riboswitch synthesis
13-1-R	CTTGTTGCCTCCTTAGCAGGGTGCTGCCAAGGGCATCAAGACGATG	
Cre-13-F	GATAACAAGATACTGACGTCGGTACCGGTGATACCAGCATCG	pSF-013 plasmid construction
Cre-13-R	TTAATCAATTCACTTCCCATCTTGTTGCCTCCTTAGCAGGG	
13-ZT-F	ATGGGAAGTGAATTGATTAAAGAAAATATGCATATG	
13-ZT-R	GACGTCAGTATCTTGTTATCCGCTCACAATG	
14-1-F	AAGTGATACCAGCATCGTCTTGATGCCCTTGGCAGCACTTCAGAAATCTCTGAAG	No. 19 theophylline riboswitch synthesis
14-1-R	CATTAACCTCCTAAAAAAAACAGCACTTCAGAGATTTCTGAAGTG	
Cre-14-F	GATAACAAGATACTGACGTCAAGTGATACCAGCATCGTCT	pSF-014 plasmid construction
Cre-14-R	TTAATCAATTCACTTCCCATCATTAACCTCCTAAAAAAAACAGCAC	
14-ZT-F	ATGGGAAGTGAATTGATTAAAGAAAATATGCATATG	
14-ZT-R	GACGTCAGTATCTTGTTATCCGCTCACAATG	
15-1-F	AATTTCATAGTTAGATCGTGTTATATGGTGAAGATAATACCAGCTTCGAA	No. 23 theophylline riboswitch synthesis
15-1-R	TTATGAACAACGAGATACTGCCAAGGGCTTCTTTCGAAGCTGGTATTATCTTCACCA	
15-2-F	CCTTGGCAGTATCTCGTTGTTCATAATCATTTATGATGATTAATTGATAAGCAATGA	
15-2-R	AAAAAAAGCAATGAGAGGAATACTCTCATTGCTTATCAATTAATCATCA	
15-3-F	TCCTCTCATTGCTTTTTTTATTGTGGACAAAGCGCTCTTTCTCCTCACCCGCACGAACC	
15-3-R	GTATTACCACCCTTTACATTTTGGTTCGTGCGGGTGAGGAGAAAGAG	
Cre-15-F	GATAACAAGATACTGACGTCAATTTCATAGTTAGATCGTGTTATATGGTG	pSF-015 plasmid construction
Cre-15-R	AATCAATTCACTTCCCATGTATTACCACCCTTTACATTTTGGTTCGTGCGGGTGAGGAG	
15-ZT-F	ATGGGAAGTGAATTGATTAAAGAAAATATGCATATG	
15-ZT-R	GACGTCAGTATCTTGTTATCCGCTCACAATG	
16-1-F	CTTCCTGACACGAAAATTTCATATCCGTTCTTAATACCAGCTTCGAAAGAAGCCCTT	No. 24 theophylline riboswitch synthesis
16-1-R	TTTTGTCTCTTCTTACTGCCAAGGGCTTCTTTCGAAGCTGGTATTAAGAACGGATAT	
16-2-F	GGCAGTAAGAAGAGACAAAATCACTGACAAAGTCTTCTTCTTAAGAGGACTTTTTTT	
16-2-R	CTTTATTCACATCAGCAAGGAAAAAAGAGAAATAAAAAAAGTCCTCTTAAGAAGAAGAC	
Cre-16-F	GATAACAAGATACTGACGTCCTTCCTGACACGAAAATTTCATATC	pSF-016 plasmid construction

Cre-16-R	TTAATCAATTCACTTCCCATTGTCTGCCTCCTTTATTCACATCAGCAAGG	
16-ZT-F	ATGGGAAGTGAATTGATTAAAGAAAATATGCATATG	
16-ZT-R	GACGTCAGTATCTTGTTATCCGCTCACAATG	
17-1-F	CAAAAAATTAATAACATTTTCTCTTATACCAGCTTCGAAAG	
17-1-R	AAAACACTGCCTCTCTCCTGCCAAGGGCTTCTTTCGAAGCTGGTATAAGAGAAAATGT	
17-2-F	AAGCCCTTGGCAGGAGAGAGGCAGTGTTTTACGTAGAAAAGCCTCTTTCTCTCATGGG	No. 25 theophylline riboswitch synthesis
17-2-R	AAAAAGCCTCTTTCCCATGAGAGAAAGAGGCTTTTCTACGT	
17-3-F	TCATGGGAAAGAGGCTTTTTGTTGTGAGAAAACCTCTTAGCAGCCTGTATCCGCGGGT	
17-3-R	TGTTTCTCCTCCTTTATATGTAAAACACTCTCTTTCACCCGCGGATACAGGCTGCTAAG	
Cre-17-F	GATAACAAGATACTGACGATCAAAAAATTAATAACATTTTCTCTTATACCAGC	
Cre-17-R	TTAATCAATTCACTTCCCATTGTTTCTCCTCCTTTATATGTAAAACACTCTC	pSF-017 plasmid construction
17-ZT-F	ATGGGAAGTGAATTGATTAAAGAAAATATGCATATG	
17-ZT-R	GACGTCAGTATCTTGTTATCCGCTCACAATG	
18-1-F	AATTAAATAGCTATTATCACGATTTTATACCAGCTTCGAAAGAAGCCCTTGGCAG	
18-1-R	AATTTTGTAATCAGGATTTTCTGCCAAGGGCTTCTTTCGAAGCTGGTATAAAAT	No. 20 theophylline riboswitch synthesis
18-2-F	AAAATCCTGATTACAAAATTTGTTTATGACATTTTTTGTAATCAGGATTTTTTTT	
18-2-R	AAAAAAAATCCTGATTACAAAAAATGTCATAAACA	
Cre-18-F	GATAACAAGATACTGACGTCAATTAAATAGCTATTATCACGATTTTATACC	
Cre-18-R	TTCCCATGGAAAATTCCTCCAAAAAAAATCCTGATTACAAAAAATGTC	pSF-018 plasmid construction
18-ZT-F	GGAGGAATTTTCCATGGGAAGTG	
18-ZT-R	GACGTCAGTATCTTGTTATCCGCTCACAATG	
19-1-F	ATACGACTCACTATAGGTGATACCAGCATCGTCTTGATGCCCTTGGCAGCACCC	No. 11 theophylline riboswitch synthesis
19-1-R	CTTGTTGTTACCTCCTTTAGCAGGGTGCTGCCAAGGGCATCAAGACGATGCTGG	
Cre-19-F	GATAACAAGATACTGACGTCATACGACTCACTATAGGTGATACC	
Cre-19-R	TTAATCAATTCACTTCCCATCTTGTTGTTACCTCCTTTAG	pSF-019 plasmid construction
19-ZT-F	ATGGGAAGTGAATTGATTAAAGAAAATATGCATATG	
19-ZT-R	GACGTCAGTATCTTGTTATCCGCTCACAATG	
20-1-F	AACGGGACTCACTATAGGTACCGGTGATACCAGCATCGTCTTGATGCCCTTGGC	No. 12 theophylline riboswitch synthesis
20-1-R	CTTGTTGCCCGGCCCGCAGGGTGCTGCCAAGGGCATCAAGACGATGCTGGTATC	
Cre-20-F	GATAACAAGATACTGACGTCAACGGGACTCACTATAGGTACCGG	pSF-020 plasmid construction
Cre-20-R	TTAATCAATTCACTTCCCATCTTGTTGCCCGGCCCGCAGGG	

20-ZT-F	ATGGGAAGTGAATTGATTAAAGAAAATATGCATATG	
20-ZT-R	GACGTCAGTATCTTGTTATCCGCTCACAATG	
21-1-F	CACTGTTCGTCAAGAAAGCATCATTGTGACTGTGTAGATTGCTATT	No. 13 theophylline riboswitch synthesis
21-1-R	AGTTTGCTCCTGATCTTCTTGTAATAGCAATCTACACAGTCACAATG	
Cre-21-F	GATAACAAGATACTGACGTCCACTGTTCGTCAAGAAAGC	
Cre-21-R	TTAATCAATTCACTTCCCATAGTTTGCTCCTGATCTTCTTGTAATAGC	pSF-021 plasmid construction
21-ZT-F	ATGGGAAGTGAATTGATTAAAGAAAATATGCATATG	
21-ZT-R	GACGTCAGTATCTTGTTATCCGCTCACAATG	
22-1-F	GGTGATACCAGCATCGTCTTGATGCCCTTGGCAGCACCCT	No. 14 theophylline riboswitch synthesis
22-1-R	GTTGTTACCTCCTTAGCAGGGTGCTGCCAAGGGCATC	
Cre-22-F	GATAACAAGATACTGACGTCGGTGATACCAGCATCGTCTTG	
Cre-22-R	TTAATCAATTCACTTCCCATGTTGTTACCTCCTTAGCAGGGTG	pSF-022 plasmid construction
22-ZT-F	ATGGGAAGTGAATTGATTAAAGAAAATATGCATATG	
22-ZT-R	GACGTCAGTATCTTGTTATCCGCTCACAATG	
23-1-F	GGTGATACCAGCATCGTCTTGATGCCCTTGGCAGCACCCT	No. 15 theophylline riboswitch synthesis
23-1-R	TAAGTTACCTCCTTAGCAGGGTGCTGCCAAGGGCATC	
Cre-23-F	GATAACAAGATACTGACGTCGGTGATACCAGCATCGTCTTG	
Cre-23-R	TTAATCAATTCACTTCCCATTAAGTTACCTCCTTAGCAGGGTG	pSF-023 plasmid construction
23-ZT-F	ATGGGAAGTGAATTGATTAAAGAAAATATGCATATG	
23-ZT-R	GACGTCAGTATCTTGTTATCCGCTCACAATG	
24-1-F	GGTGATACCAGCATCGTCTTGATGCCCTTGGCAGCAC	No. 16 theophylline riboswitch synthesis
24-1-R	TAACACACCTCCTTAGCAGGGTGCTGCCAAGGGCATCAAG	
Cre-24-F	GATAACAAGATACTGACGTCGGTGATACCAGCATCGTCTTG	
Cre-24-R	TTAATCAATTCACTTCCCATTAACACACCTCCTTAGCAGGG	pSF-024 plasmid construction
24-ZT-F	ATGGGAAGTGAATTGATTAAAGAAAATATGCATATG	
24-ZT-R	GACGTCAGTATCTTGTTATCCGCTCACAATG	
25-1-F	GGTGATACCAGCATCGTCTTGATGCCCTTGGCAGCAC	No. 17 theophylline riboswitch synthesis
25-1-R	CTTGTTGACCTCCTTAGCAGGGTGCTGCCAAGGGCATCAAG	
Cre-25-F	GATAACAAGATACTGACGTCGGTGATACCAGCATCGTCTTG	
Cre-25-R	TTAATCAATTCACTTCCCATCTTGTTGACCTCCTTAGCAG	pSF-025 plasmid construction
25-ZT-F	ATGGGAAGTGAATTGATTAAAGAAAATATGCATATG	

25-ZT-R	GACGTCAGTATCTTGTTATCCGCTCACAATG	
pRP2TRTR-VF	AAGACGATGCTGGTATCACCTTTACTAATATAACATTCAGCTTCTTTTTTGT	pRP2TRTR plasmid construction
pRP2TRTR-VR	GTAAATTAGGGCATAGATAACTCGAGAAGCTTACTAGTGGTAC	pRP2TRTR plasmid construction
pMV2TRTR-VF	AAGACGATGCTGGTATCACCAACGTTATTCTTAGCACTCGCC	Pmv261-2TRTR plasmid construction
pMV2TRTR-VR	GTAAATTAGGGCATAGATAACTAGCGTACGATCGACTGCCAG	Pmv261-2TRTR plasmid construction
164-IF	GAAGCATAAAGTGTAAAGCCGTTTTAGAGCTAGAAATAGCAAGTTAAAAT	pWA164 plasmid construction
164-VF	GGCTTTACACTTTATGCTTCACTAGTATTATACCTAGGACTGAGCTAGCT	pWA164 plasmid construction
lacZ-uparm400-Fp	AGCTTTCATCAACATTAAATGTGAGC	pWA167 and pWA170 plasmids construction
lacZ-uparm400-Rp	CATCATCATCATCATCACATGACCATGATTACGGATTCACT	pWA167 and pWA170 plasmids construction

Supplementary References

1. Koch AL. 1956. The metabolism of methylpurines by *Escherichia coli.* I. Tracer studies. *J Biol Chem* 219: 181-188
2. Quandt EM, Hammerling MJ, Summers RM, Otoupal PB, Slater B, Alnahhas RN, Dasgupta A, Bachman JL, Subramanian MV, Barrick JE. 2013. Decaffeination and measurement of caffeine content by addicted *Escherichia coli* with a refactored N-demethylation operon from *Pseudomonas putida* CBB5. *ACS Synth Biol* 2: 301-307
3. Suess B, Fink B, Berens C, Stentz R, Hillen W. 2004. A theophylline responsive riboswitch based on helix slipping controls gene expression *in vivo*. *Nucleic Acids Res* 32: 1610-1614.
4. Desai SK, Gallivan JP. 2004. Genetic screens and selections for small molecules based on a synthetic riboswitch that activates protein translation. *J Am Chem Soc* 126: 13247-13254.
5. Lynch SA, Gallivan JP. 2009. A flow cytometry-based screen for synthetic riboswitches. *Nucleic Acids Res* 37: 184-192.
6. Topp S, Reynoso CMK, Seeliger JC, Goldlust IS, Desai SK, Murat D, Shen A, Puri AW, Komeili A, Bertozzi CR, Scott JR, Gallivan JP. 2011. Synthetic riboswitches that induce gene expression in diverse bacterial species. *Appl Environ Microb* 77: 2199-2199.
7. Cui WJ, Han LC, Cheng JT, Liu ZM, Zhou L, Guo JL, Zhou ZM. 2016. Engineering an inducible gene expression system for *Bacillus subtilis* from a strong constitutive promoter and a theophylline- activated synthetic riboswitch. *Microbial Cell Factories* 15: 199.
8. Borujeni AE, Mishler DM, Wang JZ, Huso W, Salis HM. 2016. Automated physics-based design of synthetic riboswitches from diverse RNA aptamers. *Nucleic Acids Res* 44: 1-13
9. Page K, Shaffer J, Lin S, Zhang M, Liu JM. 2018. Engineering riboswitches *in vivo* using dual genetic selection and fluorescence-activated cell sorting. *ACS Synth Biol* 7: 2000-2006.
10. Canadas IC, Groothuis D, Zygouropoulou M, Rodrigues R, Minton NP. 2019. RiboCas: a universal CRISPR-based editing tool for *clostridium*. *ACS Synth Biol* 8: 1379-1390.
11. Fowler CC, Brown ED, Li Y. 2008. A FACS-based approach to engineering artificial riboswitches. *Chembiochem* 9: 1906-1911.
12. Wachsmuth M, Findeiss S, Weissheimer N, Stadler PF, Morl M. 2013. *De novo* design of a synthetic riboswitch that regulates transcription termination. *Nucleic Acids Res* 41: 2541-2551.
13. Ceres P, Garst AD, Marcano-Velazquez JG, Batey RT. 2013. Modularity of select riboswitch expression platforms enables facile engineering of novel genetic regulatory devices. *ACS Synth Biol* 2: 463-472.
14. Ogawa A, Maeda M. 2008. An artificial aptazyme-based riboswitch and its cascading system in *E. coli*. *Chembiochem* 9: 206-209.
15. Topp S, Gallivan JP. 2008. Riboswitches in unexpected places--a synthetic riboswitch in a protein coding region. *RNA* 14: 2498-2503.
16. Ceres P, Trausch JJ, Batey RT. 2013. Engineering modular 'ON' RNA switches using biological components. *Nucleic Acids Res* 41: 10449-10461.
17. Beisel CL, Storz G. 2011. The base-pairing RNA Spot 42 participates in a multioutput feedforward loop to help enact catabolite repression in *Escherichia coli. Mol Cell* 41: 286-297
18. Ali MK, Li X, Tang Q, Liu X, Chen F, Xiao J, Ali M, Chou SH, He J. 2017. Regulation of inducible potassium transporter KdpFABC by the KdpD/KdpE two-component system in *Mycobacterium smegmatis. Front Microbiol* 8: 570

19. Zhou H, Zheng C, Su J, Chen B, Fu Y, Xie Y, Tang Q, Chou SH, He J. 2016. Characterization of a natural triple-tandem c-di-GMP riboswitch and application of the riboswitch-based dual-fluorescence reporter. *Sci Rep* 6: 20871
20. Rafique N, Bashir S, Khan MZ, Hayat I, Orts W, Wong DWS. 2021. Metabolic engineering of *Bacillus subtilis* with an endopolygalacturonase gene isolated from *Pectobacterium. carotovorum*; A plant pathogenic bacterial strain. *PLoS One* 16: e0256562
21. Jiang Y, Chen B, Duan C, Sun B, Yang J, Yang S. 2015. Multigene editing in the *Escherichia coli* genome via the CRISPR-Cas9 system. *Appl Environ Microbiol*. 81:2506-2514.
22. Tribe DE, 1987. Novel microorganism and method. United States Patent 4681852, **4**, 681-852.
23. Richardson EJ, Limaye B, Inamdar H, Datta A, Manjari KS, Pullinger GD, Thomson NR, Joshi RR, Watson M, Stevens MP. 2011. Genome sequences of *Salmonella enterica* serovar typhimurium, *Choleraesuis*, *Dublin*, and *Gallinarum* strains of well- defined virulence in food-producing animals. *J Bacteriol* 193: 3162-3163
24. Li X, Mei H, Chen F, Tang Q, Yu Z, Cao X, Andongma BT, Chou SH, He J. 2017. Transcriptome landscape of *Mycobacterium smegmatis*. *Front Microbiol* 8: 2505
25. Wang X, Cai X, Ma H, Yin W, Zhu L, Li X, Lim HM, Chou SH, He J. 2019. A c-di-AMP riboswitch controlling *kdpFABC* operon transcription regulates the potassium transporter system in *Bacillus thuringiensis*. *Commun Biol* 2: 151
